# 2D Projection Maps of WSS and OSI Reveal Distinct Spatiotemporal Changes in Hemodynamics in the Murine Aorta during Ageing and Atherosclerosis

**DOI:** 10.3390/biomedicines9121856

**Published:** 2021-12-07

**Authors:** Kristina Andelovic, Patrick Winter, Thomas Kampf, Anton Xu, Peter Michael Jakob, Volker Herold, Wolfgang Rudolf Bauer, Alma Zernecke

**Affiliations:** 1 Institute of Experimental Biomedicine, University Hospital Würzburg, 97080 Würzburg, Germany; 2 Experimental Physics V, University of Würzburg, 97074 Würzburg, Germany; patrick.winter@uni-greifswald.de (P.W.); peja@physik.uni-wuerzburg.de (P.M.J.); vrherold@physik.uni-wuerzburg.de (V.H.); 3 Department of MR Physics, University of Greifswald, 17489 Greifswald, Germany; 4 Diagnostic and Interventional Neuroradiology, University Hospital Würzburg, 97080 Würzburg, Germany; thomas.kampf@physik.uni-wuerzburg.de; 5 Department of Translational Research, Comprehensive Heart Failure Center (CHFC), University Hospital Würzburg, 97080 Würzburg, Germany; xu_a@ukw.de; 6 Internal Medicine I, Cardiology, University Hospital Würzburg, 97080 Würzburg, Germany; bauer_w@ukw.de

**Keywords:** atherosclerosis, mouse, 4D flow MRI, aortic arch, flow dynamics, WSS, mapping, PWV, plaque characteristics

## Abstract

Growth, ageing and atherosclerotic plaque development alter the biomechanical forces acting on the vessel wall. However, monitoring the detailed local changes in wall shear stress (WSS) at distinct sites of the murine aortic arch over time has been challenging. Here, we studied the temporal and spatial changes in flow, WSS, oscillatory shear index (OSI) and elastic properties of healthy wildtype (WT, *n* = 5) and atherosclerotic apolipoprotein E-deficient (*Apoe*^−/−^, *n* = 6) mice during ageing and atherosclerosis using high-resolution 4D flow magnetic resonance imaging (MRI). Spatially resolved 2D projection maps of WSS and OSI of the complete aortic arch were generated, allowing the pixel-wise statistical analysis of inter- and intragroup hemodynamic changes over time and local correlations between WSS, pulse wave velocity (PWV), plaque and vessel wall characteristics. The study revealed converse differences of local hemodynamic profiles in healthy WT and atherosclerotic *Apoe*^−/−^ mice, and we identified the circumferential WSS as potential marker of plaque size and composition in advanced atherosclerosis and the radial strain as a potential marker for vascular elasticity. Two-dimensional (2D) projection maps of WSS and OSI, including statistical analysis provide a powerful tool to monitor local aortic hemodynamics during ageing and atherosclerosis. The correlation of spatially resolved hemodynamics and plaque characteristics could significantly improve our understanding of the impact of hemodynamics on atherosclerosis, which may be key to understand plaque progression towards vulnerability.

## 1. Introduction

Atherosclerosis is a complex inflammatory disease, characterized by the formation of fibrofatty lesions in the intimal layer of the artery wall (atherosclerotic plaques) [1]. Ruptures or erosions of these plaques are responsible for most of the cardiovascular events in the Western world, including myocardial infarction and stroke. Although cardiovascular risk factors, such as smoking, hypertension, hypercholesteremia and diabetes, can cause an endothelial dysfunction in the whole arterial tree, plaques develop mainly at predilection sites, such as curvatures, branch points and bifurcations [2,3]. These regions are characterized by low and oscillatory wall shear stress (WSS) that cause an endothelial dysfunction, which, together with other processes, such as lipid deposition, determine the location of plaque development [4,5]. The growing plaque furthermore leads to structural and mechanical changes in the vessel wall due to vascular remodeling and narrowing, enhancing disturbed flow patterns and affecting local WSS.

Endothelial WSS is a vector quantity, which consists of different components [6]; the longitudinal WSS (longWSS) points towards the direction of the flow. A second, circumferential component perpendicular to the longWSS can be attributed to helical flow and is orientated along the circumference of the vessel (circWSS). In addition, a third component, pointing towards the center of the vessel, can be derived, which is designated radial strain (radStrain) and can be attributed to the outwards blood pressure [7]. Besides low mean shear stress, which is mainly defined by the longitudinal WSS value, oscillatory shear stress is considered a pro-atherogenic hemodynamic parameter. It is caused by temporally oscillating flow and backflow and can be described by the oscillatory shear index (OSI). Various studies indicate a link between low and oscillatory shear stress and arterial remodeling [8,9], leading to the gradual stiffening of the arteries due to increased collagen deposition [10] and fragmentation of elastic fibers [11]. This impairment of arterial compliance is a main pathophysiological feature of atherosclerosis, representing one of the earliest measurable markers of functional and structural changes during atherosclerosis development. The assessment of the arterial pulse wave velocity (PWV) with magnetic resonance imaging (MRI) enables the non-invasive characterization of arterial elasticity by measuring the speed of the pressure waves travelling through the arterial system, which is accelerated in stiffened arteries. For preclinical in vivo studies, several imaging techniques have been developed to assess the PWV globally [12] as well as at multiple local positions along the aorta [11,13,14].

To provide insight into the influence of hemodynamics on plaque progression, a detailed analysis of spatial and temporal changes in WSS and elasticity profiles over time is essential. Given local differences in plaque growth and phenotype [12,14], the spatially resolved analysis of WSS, OSI and elasticity changes is of great interest; however, extracting these parameters in reasonable scan times remains challenging. Clinical studies demonstrated the capability of 4D flow MRI to spatially resolve WSS components in humans [15] and pixel-wise analyses have been used to detect differences between healthy subjects and Marfan patients [16]. However, preclinical applications in mice are still sparse due to technical limitations. In order to reduce errors caused by intra-observer variability and to minimize the burden on the laboratory animal, the assessment of both WSS and elasticity values in only one experimental session is desirable. In recent studies, 2D flow measurements [17], sophisticated electrocardiogram-triggered spiral and cartesian 4D flow MRI [18,19] and accelerated radial 4D flow MRI [20] have been introduced for applications in mice. However, these techniques only allow the assessment of flow or the WSS, while a simultaneous measurement of flow, WSS and PWV could only be achieved in 2D [21]. Furthermore, most studies only focus on one single animal group and one measurement time point. One of the first studies describing the simultaneous assessment of WSS and elastic properties in preclinical models using 4D flow MRI was conducted by Wentland et al., who investigated atherosclerotic lesions in pigs with familial hypercholesterolemia [22]. Recently, we demonstrated that retrospectively gated 4D PC-MRI can also be used to simultaneously extract various parameters, including the PWV and all three components of the WSS in mice from only one 4D flow measurement [7,23,24].

Here, we present a longitudinal study that uses simultaneous assessments of flow, PWV, all WSS components and the OSI in the aortic arch, based on 4D phase contrast MRI (PC-MRI) [7,23] for studying global and local hemodynamic changes in ageing wildtype (WT) and atherosclerosis-prone Apolipoprotein E-deficient (*Apoe*)^−/−^ mice during plaque progression. 2D projection maps are introduced to spatially resolve and visualize the distribution and temporal changes of hemodynamic profiles in the complete aortic arch. Pixel-wise statistical analysis for intra- and intergroup comparisons [25] revealed distinct spatiotemporal changes in WSS and OSI profiles. Correlations with elastic properties indicated that the radStrain could be of potential interest for the assessment of vascular elasticity. By correlating spatially resolved WSS and OSI values with atherosclerotic plaque parameters, we identified the circWSS as a promising new marker for advanced atherosclerotic plaque size and composition. The detailed analysis of local hemodynamics in healthy and atherosclerotic vessels will be the key to further understanding of the relationship of altered hemodynamics with atherosclerosis development and progression towards plaque vulnerability.

## 2. Materials and Methods

### 2.1. Animal Handling and Experimental Design

**Figure 1:**
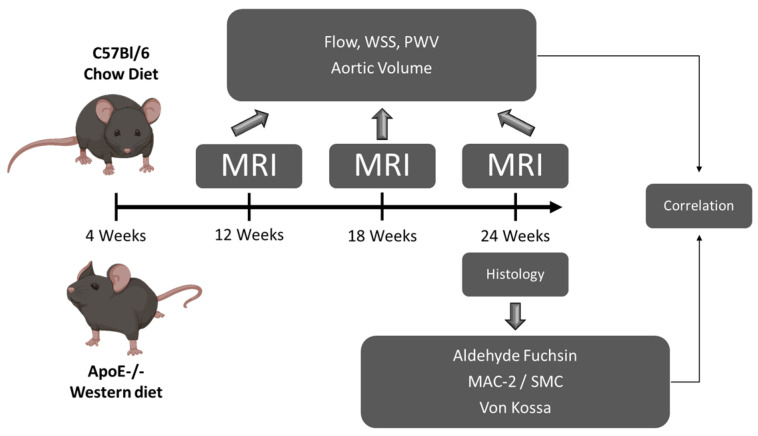
Study design. WT mice were fed a chow and *Apoe*^−/−^ mice a Western diet starting at the age of 4 weeks. At 12, 18 and 24 weeks, flow and WSS were measured with 4D-PC MRI. After 24 weeks, aortas were analyzed histologically for collagen, SMC and MAC content, necrotic core size and calcification. Obtained data were correlated with spatially resolved WSS and OSI values.

Female wildtype (WT) C57BL/6 J mice (*n* = 5) and *Apoe*^−/−^ mice (*n* = 6) (Jackson Laboratory, Bar Harbor, ME, USA) were fed a normal diet (WT) or a Western diet (*Apoe*^−/−^: ssniff, Soest, Germany) starting at the age of 4 weeks. One WT mouse died after the second measurement time point at 18 weeks due to unknown reasons. At the age of 12, 18 and 24 weeks, flow and WSS were measured using 17.6T MRI [7,23] representing early (12 weeks) [26], intermediate (18 weeks) [11] and advanced atherosclerosis (24 weeks) [27], resulting in an equal time frame of 6 weeks in between the chosen time points. Histological analyses were performed after sacrifice at the age of 24 weeks (see Figure 1). Female mice were chosen in this study to enable keeping the animals together over the duration of 20 weeks. All animal experiments were approved by local authorities (Regierung von Unterfranken, Würzburg, Germany, 18 April 2017, reference number: 55.2-2531.01-427/17) to comply with German animal protection law.

### 2.2. MRI Measurements

For MRI measurements, mice were anesthetized by applying 1.5% isoflurane in 2.0 Vol.% oxygen (2 L/min) via nose cone. A pressure-sensitive pneumatic balloon (Graseby Medical Limited, Watford, United Kingdom) was placed between the inner radio frequency resonator wall and the murine thorax to monitor vital functions (heartbeat and respiration) in real-time, by a custom-built ECG unit. Core body temperature was maintained at physiological 37 °C by adjusting the temperature of the gradient cooling unit. MRI measurements were performed using a 17.6 T vertical bore small animal MR system (Bruker Avance 750 WB, Bruker BioSpin MRI GmbH, Rheinstetten, Germany) with a 1 T/m gradient system with a diameter of 40 mm and a custom-built single-channel transmit–receive electromagnetic (TEM) resonator with an inner diameter of 24 mm. Flow was quantified in the aortic arch using a non-triggered, self-gated radial 4D-cine phase contrast sequence, as proposed recently [7,23]. All data processing and reconstructions were performed with Matlab 2016b (The Mathworks, Inc., Natick, MA, USA). Cardiac and respiratory signals were extracted from the radial MRI signal and used for retrospective 3D cine reconstruction and cardiac and breath gating, as described previously [7,23].

### 2.3. WSS Calculation

For WSS analysis, 3D cine were reconstructed at high spatial (100 µm isotropic) and moderate temporal resolution (20 frames per cardiac cycle) [7]. The lumen of the aortic arch was segmented, and a centerline of the lumen segmentation was calculated using Ensight (Ansys, Inc., Cannonsburg, PA, USA). Subsequently, the vectorial WSS $\vec{\tau }$ was derived from the 3D velocity gradients at the vessel wall, as described in [7]:

(1) $$\vec{\tau }=2\eta \dot{\varepsilon }\vec{n}$$

where $\vec{n}$ denotes the lumen surface normal vector, η denotes the blood viscosity and $\dot{\varepsilon }$ denotes the 3 × 3 deformation tensor; as follows:

(2) $${\dot{\varepsilon }}_{ij}=\frac{1}{2}\left(\frac{\partial v_{i}}{\partial x_{j}}+\frac{\partial v_{j}}{\partial x_{i}}\right),$$

where $v_{i,j}$ (*i*, *j =* 1,2,3) are the three velocity components and $x_{i,j}$ are the spatial coordinates. For the blood viscosity, a value of *η* = 0.004 Pa·s was assumed [21]. For the subsequent analysis, the WSS values were temporally averaged over the cardiac cycle.

#### Calculation of 2D Projection Maps

**Figure 2:**
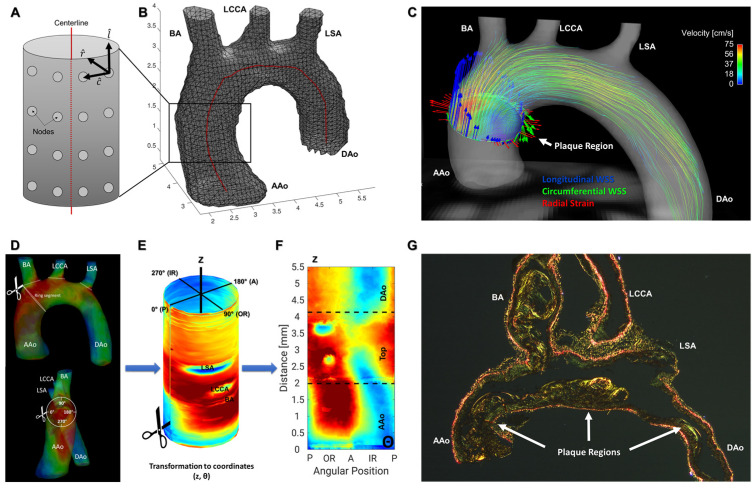
Lumen surface grid and 2D projection map generation. (**A**) Schematic zoom of an aortic segment from (**B**). For each node, WSS components ($\hat{l}$—longitudinal; $\hat{c}$—circumferential; $\hat{r}$—radial) were determined. (**B**) Lumen surface grid of the aorta with centerline (red). (**C**) Aortic arch of an *Apoe*^−/−^ mouse with streamlines and WSS components. Blue arrows: Longitudinal WSS. Green arrows: Circumferential WSS. Red arrows: Radial strain. (**D**) Anterior (top) and frontal (bottom) view of the aorta with mean WSS values and a schematic ring segment. (**E**) Branches are cut off, aorta was opened on the posterior side at 0° and the WSS was projected to the coordinates (z, θ). Convention: θ = 0°: Posterior side (P). (**F**) 2D projection map of WSS values of the arch. WSS and OSI values of the exact plaque position in the inner radius were used for histological correlation (**G**). AAo: Ascending aorta; BA: brachiocephalic artery; LCCA: left common carotid artery; LSA: left subclavian artery; DAo: descending aorta.

To separate both WSS components and the radStrain, the averaged lumen segmentation, its centerline and the temporal averaged WSS vector were used (see Figure 2B). For each node on the lumen surface grid obtained from the segmentation, the component pointing towards the centerline (radStrain), in parallel to the centerline (longWSS) and perpendicular to the first two components (circWSS) were determined [7] (Figure 2A–C).

For generation of 2D projections of the 3D WSS maps, each data WSS point (x,y,z) on the lumen surface grid was transformed into coordinates (z, θ), where z is the position on the centerline (mm), relative to the beginning of the centerline (proximal ascending aorta) and θ the angle. Afterwards, WSS components were interpolated on a WSS (z, θ) map (see Figure 2D,F). The chosen convention was θ = 90° for the outer radius (OR) of the aorta and θ = 270° for the inner radius (IR) of the aorta. Therefore, θ = 0° marks the posterior side (P) and θ = 180° the anterior side (A) (Figure 2D,E). The grid size of the interpolated maps was 0.5° in the angular direction and 1µm in longitudinal direction.

The calculation of 2D projection maps enabled a pixel-wise analysis of the WSS distribution for each individual animal as well as all groups and timepoints. For the subsequent statistical analysis, the group mean value (averaged over all animals corresponding to a specific group and time point), and the corresponding standard deviation and *p*-value was calculated for each pixel. Afterwards, 2D difference maps (e.g., control–diseased) as well as maps of the statistical significance of these differences (*p*-value), similar to those described in [16], were computed for each parameter. In the significance maps, *p*-values of *p* < 0.05 are marked in red and values of *p* > 0.05 are marked in blue, respectively. Values obtained from the 2D projection maps were correlated with PWV measurements and histological findings (Figure 2G).

### 2.4. Calculation of the Oscillatory Shear Index (OSI)

The oscillatory shear index (OSI) is a marker for temporally varying shear stress values, therefore indicating oscillating flow and backflow. It is derived from the time-dependent wall shear stress vector, $\vec{\tau }\left(t\right)$, using the following relation:

(3) $$\mathrm{OSI}\;\left[\%\right]=\frac{1}{2}\left(1-\frac{\left|\sum_{i=1}^{n}\vec{\tau }\left(i\right)\right|}{\sum_{i=1}^{n}\left|\vec{\tau }\left(i\right)\right|}\right)\cdot 100$$

Here, $\sum_{i=1}^{n}$ indicates a summation over all time frames with *n* = 20 and i=1, 2, …, 20 the frame index. The *OSI* is minimal when the WSS does not change direction or magnitude over time. On the other hand, the OSI reaches its maximum value when strong periodical variations or even sign changes occur [7].

### 2.5. PWV Analysis

PWV was estimated with the multiple-points transit-time method [12,21]. The same 4D flow data set was reconstructed at lower spatial (147 µm isotropic) and higher temporal resolution (200 frames per heart cycle). Through-plane flow was determined at approximately 50 equidistant locations along the aortic arch, as recently proposed [23]. The time point of the systolic upstroke of the volume flow was identified for each plane by measuring the intersection of a line fitted to the upstroke of the early systolic pulse and one line to the pre-systolic data points (baseline). Subsequently, the plane positions Δx (relative to the proximal ascending aorta) were plotted against the time points of the systolic upstrokes Δt. A line was fitted to the plot for PWV calculation and the PWV was afterwards derived from the slope of this fit, as follows:

(4) $$\mathrm{PWV}=\frac{\Delta x}{\Delta t}$$

For the PWV measurements at 18 weeks, we incorporated results that were originally published in [23].

### 2.6. Aortic Volume Quantification

To determine the aortic volume, the time dependent lumen segmentations of the 4D flow measurement were used. First, all segmentations were normalized to the same length by defining the center between the brachiocephalic artery and the left subclavian artery as a landmark (Figure 2B). Starting from this orientation point and using the centerline as length measure, all aortas were cut to a total length of 7 mm (3 mm along the centerline towards the proximal ascending aorta and 4 mm towards the distal descending aorta). Subsequently, the time-dependent aortic volumes were determined for all time frames by summation of the segmented voxels. The three aortic branches were excluded in order to reduce the error of the volume calculations. For the later analysis, the temporal mean, maximum and minimum values, as well as the difference between maximum and minimum volumes, were examined.

### 2.7. Atherosclerotic Lesion Quantification

Aortas were removed, cleaned from fat and connective tissue and fixed in 4% PFA overnight, embedded in paraffin and cut longitudinally into 5 µm sections. Sections from the center of the inner curvature of aortic arch (corresponding to 270° in the corresponding WSS maps) were used for further analysis. For the assessment of plaque size and collagen content, sections were stained with Gabe’s aldehyde fuchsin. In brief, sections were deparaffinized, rehydrated and stained for 15 min in aldehyde fuchsin solution to visualize elastic fibers. After 5× dipping in 70% Ethanol (EtOH), collagen was stained with picrosirius red solution for 90 min. For calcification analysis, adjacent sections were stained with Silvering after KOSSA (Morphisto, Offenbach am Main, HE, Germany) according to the manufacturer’s protocol. After dehydration, sections were embedded with Vectamount mounting medium (Vector laboratories, Burlingame, CA, USA). Immunofluorescence staining of macrophages by Mac-2 (rat anti-mouse, CL8942AP, Cedarlane, Burlington, ON, Canada) and smooth muscle cells (SMCs) by aSMA (mouse anti-mouse, C6198, Sigma) were used to analyze plaque cellular content. After heat-mediated antigen retrieval (pH = 6), slides were blocked with 5% goat serum (Sigma Aldrich), incubated with primary antibody overnight at 4 °C, followed by secondary antibody Alexa Fluor 488-conjugated antibody (Molecular Probes, Life Technologies, Germany). Sections were mounted with DAPI-containing Vectashield mounting medium (Vector laboratories, Burlingame, CA, USA). Images were taken with a Leica DM 4000B fluorescence microscope and JVC KY-F75U camera. Plaque position, size, collagen content and cellular content were quantified by computerized image analysis (Diskus Software, Hilgers, Königswinter, NRW, Germany).

For correlations of histological findings with the MRI measurements, the WSS, radStrain and OSI values were assessed from the 2D projection maps using a strip located along the inner radius of the aortic arch (location: (270 ± 30)°). Spatial averages were computed for the corresponding plaque region in the inner curvature of the ascending and descending aorta and the top region, respectively.

### 2.8. Statistical Analysis

Data are presented as mean ± SEM. All error and statistical analyses were performed in Matlab 2016b (The Mathworks, Inc., Natick, MA, USA) and GraphPad Prism 8 (GraphPad Software, San Diego, CA, USA). Outlier exclusion was performed by using the Grubbs’ test. Normal distribution was tested with the Shapiro–Wilk normality test. For normally distributed data, an unpaired t-test or an ANOVA was performed. When normality test failed, a non-parametric Mann–Whitney U test or a Kruskal–Wallis test was performed. For analysis of the degree of correlation, the Pearson correlation coefficients were calculated. For statistical analysis of the 2D projection maps, the difference values (e.g., *Apoe*^−/−^—WT) and the corresponding *p*-values were derived for each point (z, θ). Differences with *p* < 0.05 were considered statistically significant. In addition, a pixel-wise computation of the inter animal standard deviation was performed. As measure of accuracy, the spatial median as well as the lower and upper quartile values of the standard deviations were calculated.

## 3. Results

### 3.1. Aortic Volume Decreases and Aortic Flow and Pulse-Wave Velocity Increases in Atherosclerotic Apoe^−/−^ Mice

Weight, heart rates, aortic volumes and through-plane flow were assessed in 12-, 18- and 24-week-old wildtype (WT) and *Apoe*^−/−^ mice. While the weight of WT mice increased over time, no significant changes were observed in *Apoe*^−/−^ mice, with no significant differences between the groups (Supplementary Figure S1A). Heart periods did not change in either WT or *Apoe*^−/−^ mice over time, but *Apoe*^−/−^ mice featured longer heart periods compared to WT mice at 12 weeks (Supplementary Figure S1B).

**Figure 3:**
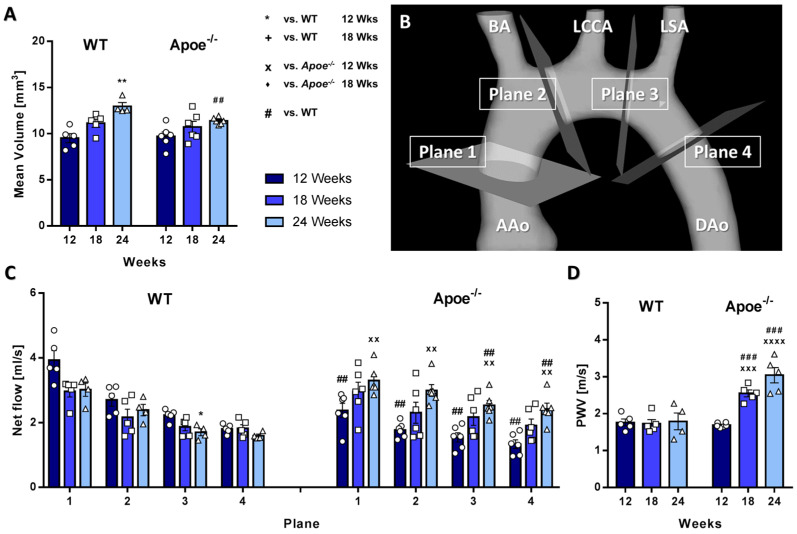
Aortic volumes are decreased and aortic flow and PWV are increased in atherosclerotic *Apoe*^−/−^ mice. (**A**) Mean aortic volumes were significantly larger in WT compared with *Apoe*^−/−^ mice at 24 weeks. (**B**) Scheme of the analyzed planes. Plane 1: Inflow into the ascending part of the aortic arch. Plane 2: Residual flow after the BA. Plane 3: Residual flow after the LCCA. Plane 4: Residual flow after the LSA. (**C**) Net flow in the 4 analysis planes. WT mice showed a decrease, whereas *Apoe*^−/−^ mice showed an increase in net flow over the course of the study. (**D**) PWV values. In contrast to WT mice, *Apoe*^−/−^ mice displayed an increase in PWV over time. * vs. WT 12 weeks, *p* < 0.05; ** vs. WT 12 weeks, *p* < 0.01; ^+^ vs. WT 18 weeks, *p* < 0.05; ^x^ vs. *Apoe*^−/−^ 12 weeks, *p* < 0.05; ^xx^ vs. *Apoe*^−/−^ 12 weeks, *p* < 0.01; ^xxx^ vs. *Apoe*^−/−^ 12 weeks, *p* < 0.001; ^xxxx^ vs. *Apoe*^−/−^ 12 weeks, *p* < 0.0001; ^♦^ vs. *Apoe*^−/−^ 18 weeks, *p* < 0.05; ^#^ vs. WT, *p* < 0.05; ^##^ vs. WT, *p* < 0.01; ^###^ vs. WT, *p* < 0.001.

Both mouse groups showed an increase in aortic volumes over time; however, 24-week-old *Apoe*^−/−^ mice featured significantly smaller mean volumes compared with the control group (Figure 3A). Maximum (systolic) and minimum (diastolic) aortic volumes were also significantly smaller in atherosclerotic *Apoe*^−/−^ mice compared with WT mice (Supplementary Figure S1). Furthermore, WT mice showed a stronger increase in maximum and minimum aortic volumes compared with *Apoe*^−/−^ mice over time (Supplementary Figure S1C,D). In line, differences between systolic and diastolic volumes were smaller compared with WT mice, indicating an impaired dilatability during atherosclerotic plaque progression (Supplementary Figure S1). Net flow over the cardiac cycle increased over time in *Apoe*^−/−^ but remained constant in WT mice (Figure 3B,C). Consistently elevated flow values were found in atherosclerotic mice at 24 weeks. Peak flow over the cardiac cycle was quantified in four analysis planes (Figure 3B). In line with the net flow, a trend towards decreased peak flow along the aorta was observed. In WT mice, no significant changes in peak flow values were noted over time (Supplementary Figure S1F). In *Apoe*^−/−^ mice, however, peak flow significantly increased over time, resulting in significant differences in analysis planes 2–4 (Supplementary Figure S1F). While no significant changes in PWV values were observed in WT mice over time, PWV increased in *Apoe*^−/−^ mice exhibiting significant differences, indicative of aortic stiffening (Figure 3D).

### 3.2. Longitudinal WSS Increases in Apoe^−/−^ mice during Atherosclerosis Progression but Decreases in WT Mice over Time

**Figure 4:**
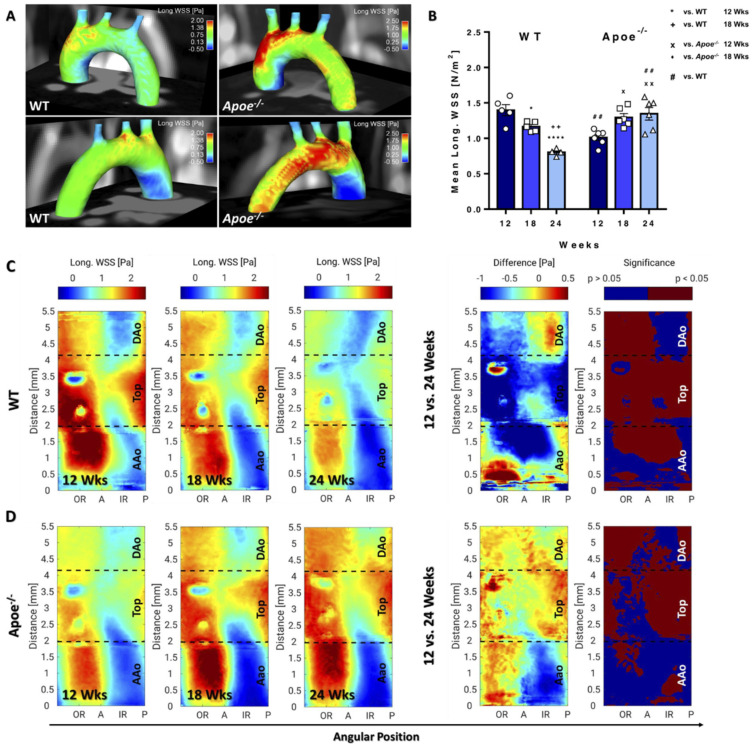
LongWSS increases in *Apoe*^−/−^ mice during atherosclerosis progression but decreases in WT mice over time. (**A**) Three-dimensional longWSS map of a 24-week-old WT (left) and *Apoe*^−/−^ mouse (right) from anterior and posterior view. A stronger WSS gradient was observed in *Apoe*^−/−^ compared with WT mice and in the IR, lowered WSS is observable (dark blue spots). (**B**) Mean longWSS was decreasing in WT and increasing in *Apoe*^−/−^ mice over time. * vs. WT 12 weeks, *p* < 0.05; **** vs. WT 12 weeks, *p* < 0.0001; ^++^ vs. WT 18 weeks, *p* < 0.01; ^x^ vs. *Apoe*^−/−^ 12 weeks, *p* < 0.05; ^xx^ vs. *Apoe*^−/−^ 12 weeks; ^#^ vs. WT, *p* < 0.05; ^##^ vs. WT, *p* < 0.01. (**C**) LongWSS maps of WT mice (group average) for all measurement time points and statistical intragroup comparison of longWSS values (12 vs. 24 weeks), showing the difference maps of longWSS values and significance maps. (**D**) LongWSS Maps of *Apoe*^−/−^ mice.

The spatial mean and maximum values provide global information on WSS differences in the aortic arch. However, both the development of plaques and WSS locally affect the aorta, revealing strong differences of longWSS in the 3D visualization, in particular in the ascending aorta (AAo) and posterior side of the aorta, when comparing WT with *Apoe*^−/−^ mice, where a much stronger WSS gradient was observed (Figure 4A). In WT mice, a continuous, significant decrease in spatial mean and maximum longWSS was measured over time. Mean (Figure 4B) and maximum (Supplementary Figure S2A) values were significantly lower in *Apoe*^−/−^ compared with WT mice, but a significant increase was observed during atherosclerosis progression. As a result, 24-week-old *Apoe*^−/−^ mice featured significantly higher values compared with respective WT mice, suggesting an increased WSS due to elevated flow, plaque growth and lumen narrowing.

Spatially resolved WSS values were visualized by generating 2D projection maps of the whole aortic arch for each time point. In all measurements, an asymmetric distribution with highest values near the outer radius (OR) and lowest values near the inner radius (IR) was observed (Figure 4C,D). For error estimation, the spatial median values of the inter animal standard deviations were calculated for all groups and timepoints. The results indicate maximum median values of 0.34Pa and 0.39Pa in WT and *Apoe*^−/−^ mice, respectively (see bold values in Supplementary Tables S1 and S2), and upper quartile values of 0.45Pa and 0.54Pa, respectively. The subsequent statistical analysis showed a significant decrease in longitudinal WSS values throughout the aorta of WT mice over time, leading to a decreased WSS gradient when comparing OR and IR (Figure 4C). In atherosclerotic *Apoe*^−/−^ mice, however, a significant increase in longWSS in the top region and descending aorta (DAo) and a significant decrease around the IR of the AAo was observed (Figure 4D), resulting in an increasing WSS gradient over time (Figure 4D). When comparing both mouse models, *Apoe*^−/−^ mice feature significantly lower longWSS mostly in the AAo and top region at 12 weeks and show gradually increased values over time and significantly higher longWSS in the top region and descending aorta (DAo) as well as in the OR of the AAo at 24 weeks, confirming a stronger WSS gradient compared with WT mice (Supplementary Figure S3).

### 3.3. Circumferential WSS Shows Inverse Changes in Apoe^−/−^ and WT Mice, Whereas Radial Strain Only Shows Local Changes

**Figure 5:**
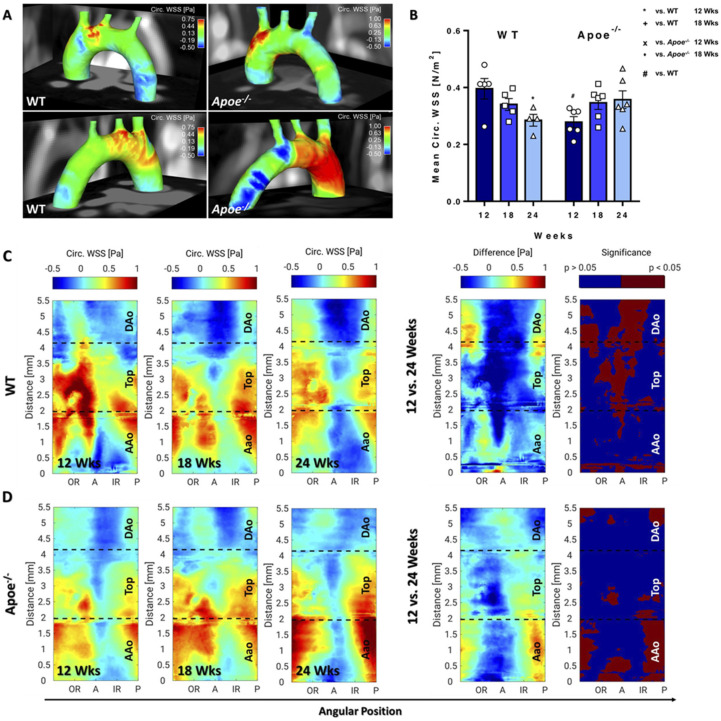
CircWSS shows inverse changes in *Apoe*^−/−^ and WT mice. (**A**) Three-dimensional circWSS map of a 24-week-old WT mouse (left) and an *Apoe*^−/−^ mouse (right) from anterior and posterior view. In comparison with the WT, high circWSS is visible in the posterior side of the AAo in the *Apoe*^−/−^ mouse (red area in bottom right image). (**B**) Mean circWSS. WT mice showed significantly higher values at 12 compared to 24 weeks and compared with age-matched *Apoe*^−/−^ mice. * vs. WT 12 weeks, *p* < 0.05; ^#^ vs. WT, *p* < 0.05. (**C**) CircWSS maps of WT mice (group average) for all measurement time points and statistical intragroup comparison of circWSS values (12 vs. 24 weeks), showing the difference maps of circWSS values and significance maps. (**D**) CircWSS maps of *Apoe*^−/−^ mice.

The 3D visualization of circWSS revealed high values around the posterior side of the AAo in the *Apoe*^−/−^ mouse in comparison with WT mice (Figure 5A). Mean circWSS showed a similar pattern as longWSS in WT mice, decreasing significantly over time (Figure 5B). In *Apoe*^−/−^ mice, a non-significant trend towards increased mean circWSS, and a significant increase in maximum values were observed (Figure 5B and Figure S4). While *Apoe*^−/−^ mice showed a decreased circWSS at 12 weeks compared with WT mice, both groups featured similar mean circWSS values at 18 and 24 weeks (Figure 5B and Figure S4). At 24 weeks, however, maximum values were elevated in *Apoe*^−/−^ relative to WT mice (Supplementary Figure S4). 2D projection maps of WT mice revealed a significantly decreasing circWSS around the anterior region and IR of the top and descending aorta during ageing (Figure 5C). In *Apoe*^−/−^ mice, only mild changes were found during atheroprogression, with significantly increased values in the IR of the AAo, indicative of plaque growth (Figure 5D). Compared with WT mice, the most significant changes were noted at 24 weeks (Supplementary Figure S4), with increased values in the IR and posterior side of the AAo, and between the anterior and IR of the DAo in *Apoe*^−/−^ mice, suggestive of increased helical flow in this area. In contrast, circWSS values were partially decreased in the OR. A corresponding analysis of the inter animal deviations revealed maximum median values of 0.30Pa and 0.25Pa (see bold values in Supplementary Tables S1 and S2) and upper quartile values of 0.40Pa and 0.35Pa in WT and *Apoe*^−/−^ mice, respectively.

The 3D visualization of radStrain showed large areas with negative magnitude values near the outer radius of the AAo and Dao in the *Apoe*^−/−^ mice (Supplementary Figure S5A). The WT mice, on the other hand, featured a significantly different pattern and positive values around the IR of the AAo. Mean radStrain did not change over time in WT and *Apoe*^−/−^ mice, but significantly lower values were observed in 12-week-old *Apoe*^−/−^ compared with age-matched WT mice (Supplementary Figure S5B). Similarly, peak radStrain values were lower in *Apoe*^−/−^ mice and a significant decrease in peak radStrain was observed in WT mice over time (Supplementary Figures S2C and S6). In *Apoe*^−/−^ mice, no clear trend was observed (Supplementary Figure S2C). However, local changes of radStrain were revealed by the 2D projection maps. In WT mice, a steady increase in radStrain was observed around the OR of the top and descending aorta, and a decrease around the IR of the same region (Supplementary Figure S5C). In contrast, *Apoe*^−/−^ mice featured significantly decreased values around the IR of the AAo and top region, indicative of arterial stiffening (Supplementary Figure S5C). Comparisons of WT and *Apoe*^−/−^ mice revealed drastic local changes of radStrain throughout the complete aortic arch during the course of the study (Supplementary Figure S6). In comparison with WT mice, *Apoe*^−/−^ mice featured significantly decreased values around the IR of the AAo and OR of the DAo. A corresponding increase was observed around the OR of the AAo and the IR of the DAo. The analysis of the inter-animal standard deviations yielded maximum median values of 0.47Pa and 0.50Pa (see bold values in Supplementary Tables S1 and S2) and upper quartile values of 0.67Pa and 0.71Pa in WT and *Apoe*^−/−^ mice, respectively.

### 3.4. OSI Decreases in Apoe^−/−^ Mice during Atherosclerosis Progression but Increases in WT Mice during Ageing

**Figure 6:**
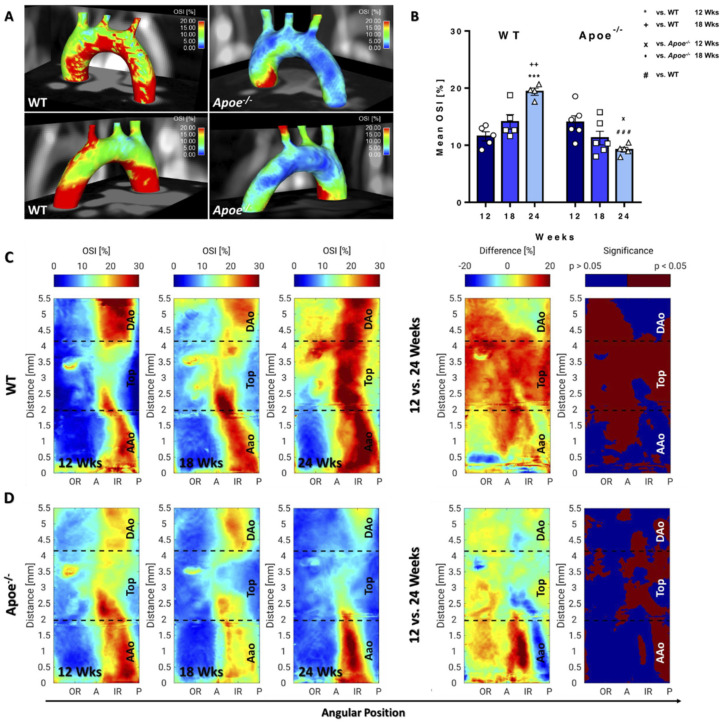
OSI decreases in *Apoe*^−/−^ mice during atherosclerosis progression but increases in WT mice during ageing. (**A**) Three-dimensional OSI map of a 24-week-old WT mouse (left) and *Apoe*^−/−^ mouse (right) from anterior and posterior view. The WT mouse showed high OSI values throughout the IR of the complete aorta, whereas in the *Apoe*^−/−^ mouse, values were only elevated in the IR of the AAo (see red spot). (**B**) Mean OSI: Values significantly increased in WT and decreased in *Apoe*^−/−^ mice. *** vs. WT 12 weeks, *p* < 0.001; ^++^ vs. WT 18 weeks, *p* < 0.01; ^x^ vs. *Apoe*^−/−^ 12 weeks, *p* < 0.05; ^###^ vs. WT, *p* < 0.001. (**C**) OSI maps of WT mice (group average) for all measurement time points and statistical intragroup comparison of OSI values (12 vs. 24 weeks), showing the difference maps of OSI values and significance maps. (**D**) OSI maps of *Apoe*^−/−^ mice.

3D visualization of OSI values revealed elevated OSI values throughout the IR of the aorta in WT mice, whereas in *Apoe*^−/−^ mice, elevated levels were only found in the IR of the AAo (red spot, Figure 6A). Mean OSI increased in WT mice over time, reaching high statistical significance at 24 compared with 12 weeks (Figure 6B). Conversely, a decrease in mean OSI was noted in *Apoe*^−/−^ mice with significant changes after 24 weeks, leading to significantly lower values compared with WT mice. However, no differences in maximum OSI values were noted between the genotypes and time-points (Supplementary Figure S2D). 2D projection maps demonstrated that the OSI increased in large areas throughout the aortic arch in WT mice over time (Figure 6C), in particular in the outer top region of the arch, extending to the DAo at 24 weeks. In contrast, *Apoe*^−/−^ mice showed significantly decreased OSI values around the IR and posterior side of the complete aortic arch over time (Figure 6D). Comparing WT and *Apoe*^−/−^ mice, OSI values were regionally increased in *Apoe*^−/−^ mice at 12 weeks and significantly decreased in the complete top and DAo. However, in the AAo, a significant, regional decrease was found around the posterior and anterior region (Supplementary Figure S7). The corresponding analysis of the inter animal standard deviations resulted in maximum median values of 5.8% and 6.3% (see bold values in Supplementary Tables S1 and S2) and respective upper quartile values of 8.4% and 7.8% in WT and *Apoe*^−/−^ mice, respectively.

### 3.5. Plaque Characteristics Correlate with WSS

**Figure 7:**
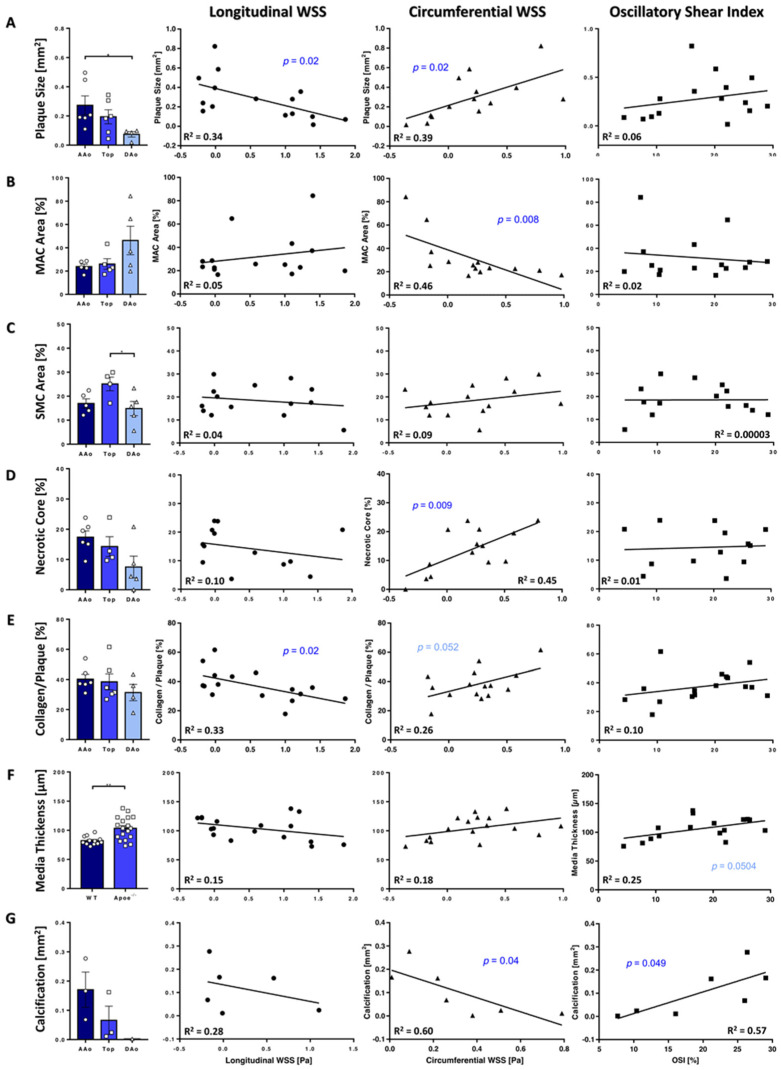
Plaque characteristics correlate with WSS values. (**A**) Plaque size was significantly smaller in the DAo compared with the AAo (*p* < 0.05, *). A correlation was found with longWSS and circWSS. (**B**) Relative MAC area. A correlation with circWSS was observed. (**C**) SMC area in atherosclerotic plaques. In the top region, a significantly higher SMC content was observed compared with the DAo (*p* < 0.05, *), but no correlation with WSS was found. (**D**) Necrotic core area showed no regional differences but positively correlated with circWSS. (**E**) Collagen analysis in atherosclerotic plaques. A correlation with longWSS was found. (**F**) Media thickness was significantly increased in *Apoe*^−/−^ mice (*p* < 0.01, **), but no significant correlation with WSS was found. (**G**) Calcification: Larger areas were found in the AAo. A negative correlation was found with circWSS and a positive correlation with OSI.

WSS may have distinct and different impact on atherosclerotic lesion formation and growth; therefore, we examined lesion size and characteristics in *Apoe*^−/−^ mice upon sacrifice after 24 weeks (Supplementary Figure S8) in correlation with WSS and OSI values. Plaque size was significantly smaller in the DAo compared with the AAo and negatively correlated with longWSS values. An increase in plaque size was observed with increasing circWSS values, but no correlations were found with OSI values (Figure 7A). Analysis of the plaque macrophage content revealed a tendency towards higher relative ratios in the DAo compared with the AAo and negatively correlated with circWSS (Figure 7B). Smooth muscle cell (SMC) content was higher in the top region compared with the DAo, but no correlations of SMC content with WSS or OSI was detected (Figure 7C). Relative necrotic core area showed no regional differences, but a positive correlation with circWSS (Figure 7D).

Plaque collagen content showed no area-specific differences, but a negative correlation with longWSS was observed (Figure 7E). For circWSS, however, a trend towards higher plaque collagen content with increasing WSS was visible. Media thickness was significantly increased in *Apoe*^−/−^ compared with WT mice but showed no correlation with WSS values (Figure 7F). However, a positive trend towards increased media thickness with higher OSI values was noticeable. Calcified plaque regions were found in the AAo and top region but not in the Dao and we observed a negative correlation with circWSS and a positive correlation with the OSI (Figure 7G).

### 3.6. PWV Correlates with WSS and Vessel Wall Characteristics

**Figure 8:**
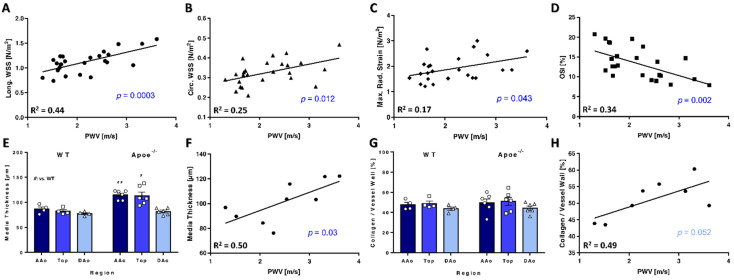
PWV correlates with WSS and vessel wall characteristics. (**A**) Correlation with longWSS. (**B**) Correlation with circWSS. (**C**) Correlation with maximum radStrain. (**D**) Correlation with OSI. (**E**) Media thickness: Significantly higher values were found in the AAo and top region of *Apoe*^−/−^ compared with WT mice. (**F**) Correlation with media thickness. Media thickening increases PWV. (**G**) Collagen content of the vessel wall. No differences were observed. (**H**) Correlation with collagen content in the vessel wall. ^#^ vs. WT, *p* < 0.05; vs. WT, ^##^ *p* < 0.01.

The development of large plaques throughout the complete aortic arch, leading to a loss of elasticity [11] and a constriction of the aortic lumen may result in increased PWV and longWSS values. Correlative analyses indeed demonstrated an increase in longWSS and circWSS with increasing PWV in all mice (Figure 8A,B). Furthermore, a significant correlation of maximum radStrain and PWV was observed (Figure 8C). In contrast, increasing OSI values negatively correlated with PWV (Figure 8D).

Media thickness showed no changes in both groups across the AAo, the top region or the DAo over time, although media thickness was significantly increased in the AAo and top region of *Apoe*^−/−^ compared with WT mice (Figure 8E). Interestingly, mean values of the media thickness correlated with PWV values (Figure 8F). Collagen content in the vessel wall was similar throughout the regions and between genotypes (Figure 8G), but a strong trend towards increased PWV values was noted in arteries with higher collagen content (Figure 8H).

## 4. Discussion

Although altered WSS, elasticity and inflammation are closely intertwined and critical for plaque development, their growth and progression towards vulnerability, preclinical investigations still mostly focus on the two-dimensional analysis of only one of these parameters. Here, we applied high-resolution 4D PC-MRI as a powerful, non-invasive imaging modality [7,23] for the simultaneous evaluation of hemodynamic profiles and elasticity in healthy WT and atherosclerotic *Apoe*^−/−^ mice. Visualization of all WSS components and OSI and the statistical evaluation of spatiotemporal changes using 2D projection maps was revealed to be a potent tool allowing their spatially resolved correlation with histological parameters.

In *Apoe*^−/−^ mice, hemodynamic profiles were evaluated during early atherosclerosis with beginning plaque formation at 12 weeks of age (corresponding to 8 weeks of Western diet) [26], intermediate atherosclerosis with measurable arterial stiffening at 18 weeks of age (corresponding to 14 weeks of diet) [11] and advanced atherosclerosis at 24 weeks of age (corresponding to 20 weeks of diet), resulting in progressive aortic atherosclerotic plaques [27]. The aging process in WT mice was observed in parallel at the same time points. The study revealed a contrary development of various hemodynamic parameters during ageing and atherosclerosis progression. Net flow was lower in *Apoe*^−/−^ mice compared with WT mice, increasing over time in the atherosclerotic mouse group, but decreasing in WT mice. Both results are in good accordance with ultrasound measurements conducted in *Apoe*^−/−^ and WT mice on normal diet [28], and in line with previous observations of elevated aortic flow in the ascending and abdominal aorta of 8 months old *Apoe*^−/−^ mice [29,30]. In addition, *Apoe*^−/−^ mice showed reduced aortic volumes, in line with prior observations [30], attributable to plaque development constricting the lumen, as additionally confirmed by histology.

Furthermore, the difference between the maximum (systolic) and minimum (diastolic) aortic volume was decreased in *Apoe*^−/−^ mice, indicating an impaired dilatability due to an increased stiffness of the aorta. This notion is also supported by the aortic PWV values, which significantly increased in *Apoe*^−/−^ mice but stayed constant in the control group, in agreement with previous results [11,31]. The PWV was shown to serve as a surrogate marker for early atherosclerotic vessel wall changes such as loss of arterial compliance due to elastin fragmentation [11]. Here, we provide evidence that PWV also correlates with media thickness and possibly collagen content, which may become even more apparent with an increased group size. Interestingly, PWV also correlated with maximum radStrain, indicating that this parameter could be a further possible marker of arterial compliance. *Apoe*^−/−^ mice revealed significantly lower local radStrain values in the AAo, attributable to increased local stiffness. Our previous work already pointed out a possible connection between radStrain and the radial dilatability of the vessel [7]. Hence, we conclude that mapping of this parameter may describe vascular elasticity locally. However, this needs further investigation and confirmation through correlations with other elasticity measurements and computational fluid dynamics (CFD) [14].

Not only elasticity is an important factor in atherosclerosis development; local shear stress conditions are also relevant to understand the connection between altered hemodynamics and atherosclerosis. However, mean WSS values only provide global information about differences between healthy and diseased mice. Generating 2D projection maps allowed us to spatially resolve and visualize information about all WSS components and OSI during ageing and atherosclerosis. Here, changes in the longitudinal WSS profile over time also showed a contrary behavior, resulting in a steadily decreasing WSS gradient during ageing, attributable to increasing aortic volumes but constant net flow and elasticity. In contrast, during atherosclerosis progression, an increasing WSS gradient in particular in the ascending aorta was observed, attributable to an increased flow through smaller and stiffer vessels, resulting in steeper flow profiles and therefore elevated WSS values in *Apoe*^−/−^ mice. These results are in agreement with values reported in previous studies [18,21]. Furthermore, another study showed a drastic increase in vessel wall thickness due to plaque development in *Apoe*^−/−^ mice, resulting in lumen narrowing and a consequent loss of arterial compliance [11], which is in line with the observed correlation of increasing longWSS and PWV. The significant drop in longWSS observed in WT mice, however, might be explained by increasing lumen volumes due to growth. Additionally, constant net flow was found in all analysis planes except plane 3, which together results in less steep flow profiles and, in consequence, lower longitudinal WSS values.

Circumferential WSS, on the other hand, significantly increased over time during plaque growth in the ascending aorta of atherosclerotic mice, where the largest plaque burden was found. In WT mice, however, the circWSS decreased globally and locally in the top and descending aorta, similar to the longWSS component, which may be connected to the increasing aortic volumes.

A well-established marker for the characterization of oscillating flow and back-flow is the OSI. High OSI values mark large temporal varying changes in these specific regions, whereas low OSI values are found in regions of unidirectional, laminar flow. Our intergroup comparison revealed significantly elevated OSI values in the AAo, top region and DAo of 12-week-old atherosclerotic mice in comparison with WT mice. In the following, however, OSI decreased in *Apoe*^−/−^ mice, and only the region near the inner curvature of the AAo featured large OSI values, where the largest plaque burden was observed. The drop of OSI values in atherosclerotic mice might thus be explained by structural changes of the vessel wall during plaque development. While longWSS increases, the fraction of temporally oscillating flow decreases, which consequently may lead to significantly lower OSI values in stiffened and constricted arteries. In WT mice on the other hand, OSI increased over time, attributable to increasing lumen volumes during ageing.

Furthermore, WSS is connected to plaque size, as revealed by correlations with histological analyses. Higher longWSS was observed in aortas with smaller plaque size, which is in line with results reported for models of cuff-induced plaque formation [32,33]. For the circWSS, a converse relation was found, supporting the assumption that low longWSS and high circWSS, attributable to high helical flow, are pro-atherogenic factors causing plaque growth and progression.

An increased collagen production is known to impact on arterial elasticity. Collagen content in the media did not differ significantly between healthy and atherosclerotic mice. However, collagen is also found in atherosclerotic plaques (20–60% collagen), which may have contributed to the increase in PWV in *Apoe*^−/−^ compared with WT mice. Interestingly, plaque collagen content showed no regional differences, but significantly smaller plaques were found in the DAo compared with the AAo and a decreased collagen content was found in areas with higher longWSS. During atherosclerosis, SMCs migrate into the intima, where they proliferate and produce extracellular matrix components, including collagen, to form the fibrous cap [34]. Furthermore, laminar shear stress was shown to inhibit [35], while oscillatory shear stress stimulates SMC proliferation [36]. A lower SMC-derived collagen content within plaques may thus be in line with increased longWSS. Importantly, increased WSS values were reported to be associated with plaque rupture [37], whereas vortices causing oscillatory shear stress induce more stable plaques with higher SMC and collagen content [32,33].

Monocytes/macrophages play key roles in the formation of atherosclerotic plaques, and monocyte recruitment is initiated through the dysfunction and activation of the vascular endothelium, a process intricately linked to local hemodynamics [38]. We noted a tendency towards lower macrophage but larger necrotic core content in the AAo compared with the DAo, in line with larger and more advanced lesions in the AAo, where atherosclerotic lesions form earlier than in the descending aorta. Interestingly, macrophages were significantly reduced in areas with high circWSS. This observation is in line with findings by Xing et al., who observed a greater accumulation of macrophages in plaques exposed to lower WSS in mice [32]. Although a high OSI environment was identified to correlate with plaque composition [32,33], and even erosion [39], we only found a correlation with plaque calcification. We cannot exclude the possibility that this could have been caused by decreased SNR in regions with high OSI and low WSS values [7].

In our study, circWSS correlated with different parameters, including plaque size, macrophage content, calcification and necrotic core area. Elevated circWSS values indicate alterations of helical flow, presumably related to the changes in the vessel wall morphology and were detected in atheroprone regions [18]. Moreover, studies in humans have demonstrated that circumferential WSS, as assessed by MRI, could serve as a parameter to study multidirectional flow in complex geometries [40,41]. We thus propose the circumferential WSS component as a potential parameter to non-invasively evaluate plaque characteristics in advanced atherosclerosis.

### 4.1. Accuracy

The reproducibility as well as the stability of the 4D flow measurement have been examined in detail in a previous study in wildtype mice, showing good reproducibility of the WSS components [7,23]. The stability of the measurement, assessed by using subsampling, revealed a measurement error in the order of 0.1 Pa for the WSS components and the radial strain [7,23]. For a further investigation of the measurement error present in this study, the inter-animal standard deviations were analyzed in both wildtype and *Apoe*^−/−^ mice (Supplementary Tables S1 and S2). We observed larger deviation values in comparison with the error of a single measurement. This is most likely due to natural variations between individual animals, differences in slice positioning, shim, etc. However, these deviations were lower than the observed inter group differences (see for example the difference map in Figure 4C, where difference values of more than 1 Pa were observable).

### 4.2. Limitations

The 2D analysis of histological sections does not reflect the 3D properties of the aortic arch and regional characteristics of the plaque. Therefore, a three-dimensional assessment of plaque characteristics, such as morphological measurements with MRI [42] or utilization of light sheet microscopy techniques [43], combined with spatially resolved WSS mapping, should be applied to further uncover the interrelationship of hemodynamics and atherosclerosis development and progression in the future. The results of this study furthermore point to a possible link between radial strain and radial aortic dilatability. In future studies, these results need to be correlated with local PWV measurements [12,14] in order to investigate if radial strain indeed is capable of spatially resolving vessel wall elasticity. More thorough investigations, e.g., through CFD, need to be conducted in the future to exclude all possible sources of measurement errors, e.g., caused by displacement artifacts [44]. A further limitation is the relatively small sample size of the animal groups. While the study presented in this work already reveals interesting results, such as the correlation of WSS with plaque size, and previous studies already demonstrated good reproducibility and stability [7,23], larger sample sizes and the investigation of earlier time points in atherogenesis will be necessary to further resolve possible correlations between plaque characteristics and OSI. Moreover, in order to prove the feasibility to quantify local differences in WSS and OSI profiles in mice, the study was conducted in female mice. Findings in humans already underline gender-specific differences in atherosclerosis, whereas conflicting results are reported in mice, as recently reviewed by Man et al. [45]. Interestingly, a recent study conducted by Crouch et al. [17] pointed out sex- and age-related differences of flow and WSS values in different locations of the murine vascular tree. Therefore, it will be important to compare female and male mice in future studies to investigate spatially resolved hemodynamics, including correlations with plaque size and composition and direct statistical comparison of the different sexes.

## 5. Conclusions and Future Perspectives

We demonstrate the feasibility and utility of high-resolution 4D flow MRI to spatially resolve (100 µm) all hemodynamic parameters in healthy and atherosclerotic mice over time, using 2D projection maps as a promising and powerful tool to enable local correlations of hemodynamics with plaque characteristics. The detailed mapping and statistical evaluation of all three WSS components and OSI in the complete aortic arch provide unprecedented insight into flow-dependent changes during the natural course of ageing and atherosclerotic disease progression and could contribute to an improved understanding of the association between altered hemodynamics and plaque progression. In future studies, early atherosclerotic lesion development could be investigated in order to reveal the relationship of altered hemodynamics and early vascular inflammation driving plaque development. Furthermore, efforts should be made to further characterize cellular plaque composition and plaque characteristics in a three-dimensional manner. The detailed, spatially resolved 3D-analysis of functional and structural plaque and vessel parameters, in combination with the spatially resolved 2D projection maps, could significantly improve preclinical atherosclerosis research. Moreover, our flexible reconstruction frameworks, developed in a preclinical application, could straightforwardly be applied to flow measurements in whole body MRI scanners [46], adapted to human applications and may even be utilized to map WSS and OSI in smaller arteries. As all hemodynamic parameters are assessed from one single measurement, an application in the clinic could be achieved in short examination times. The combined measurement of PWV and all WSS components may be a powerful tool to investigate local hemodynamics and plaque characteristics non-invasively to guide diagnosis and therapy in the future.

## Data Availability

The data presented in this study are available on request from the corresponding authors.

## Supplementary Materials

The following are available online at https://www.mdpi.com/article/10.3390/biomedicines9121856/s1. Figure S1: Weight, heart periods, aortic volumes and peak flow of wildtype and *Apoe*^−/−^ mice for all measurement time points. Figure S2: Maximum WSS and OSI values for wildtype and *Apoe*^−/−^ mice over time. Figure S3: Intergroup comparison of LongWSS for all measurement time points. Figure S4: Intergroup comparison of CircWSS for all measurement time points. Figure S5: Radial strain shows modest changes in *Apoe*^−/−^ and WT mice over time. Figure S6: Intergroup comparison of RadStrain for all measurement time points. Figure S7: Intergroup comparison of OSI for all measurement time points. Figure S8: Histological stainings of longitudinal sections of an exemplary *Apoe*^−/−^ mouse. Table S1: Spatial median, lower quartile and upper quartile values of the intragroup standard deviations (STD) in wild type mice, determined from the pixel values of the 2D projection maps. Table S2: Spatial median, lower quartile and upper quartile values of the intra group standard deviations (STD) in *Apoe*^−/−^ mice, determined from the pixel values of the 2D projection maps.

## List of Abbreviations

2D	Two-dimensional
3D	Three-dimensional
4D	Four-dimensional
A	Anterior side
AAo	Ascending Aorta
*Apoe* ^−/−^	Apolipoprotein E-deficient
BCA	Brachiocephalic artery
CircWSS	Circumferential WSS
DAo	Descending Aorta
DAPI	4‘,6-Diamidin-2-phenylindol
EtOH	Ethanol
IR	Inner radius
LCCA	Left common carotid artery
LongWSS	Longitudinal WSS
LSA	Left subclavian artery
MAC	Macrophages
MRI	Magnetic resonance imaging
OR	Outer radius
OSI	Oscillatory shear index
P	Posterior
PBS	Phosphate buffered saline
PC	Phase contrast
PFA	Paraformaldehyde
PWV	Pulse wave velocity
RadStrain	Radial strain
SD	Standard deviation
SEM	Standard error of mean
SMCs	Smooth muscle cells
WSS	Wall shear stress
WT	Wildtype
