# Supplementary material for: 2D Projection Maps of WSS and OSI Reveal Distinct Spatiotemporal Changes in Hemodynamics in the Murine Aorta during Ageing and Atherosclerosis
Source: Biomedicines. 2021 Dec 7;9(12):1856. doi: 10.3390/biomedicines9121856 (PMC8698968; doi:10.3390/biomedicines9121856)
Supplement: Supplementary file 1 [file biomedicines-09-01856-s001.zip › Supplementary Figures_Captions.pdf]

## Supplementary Figures

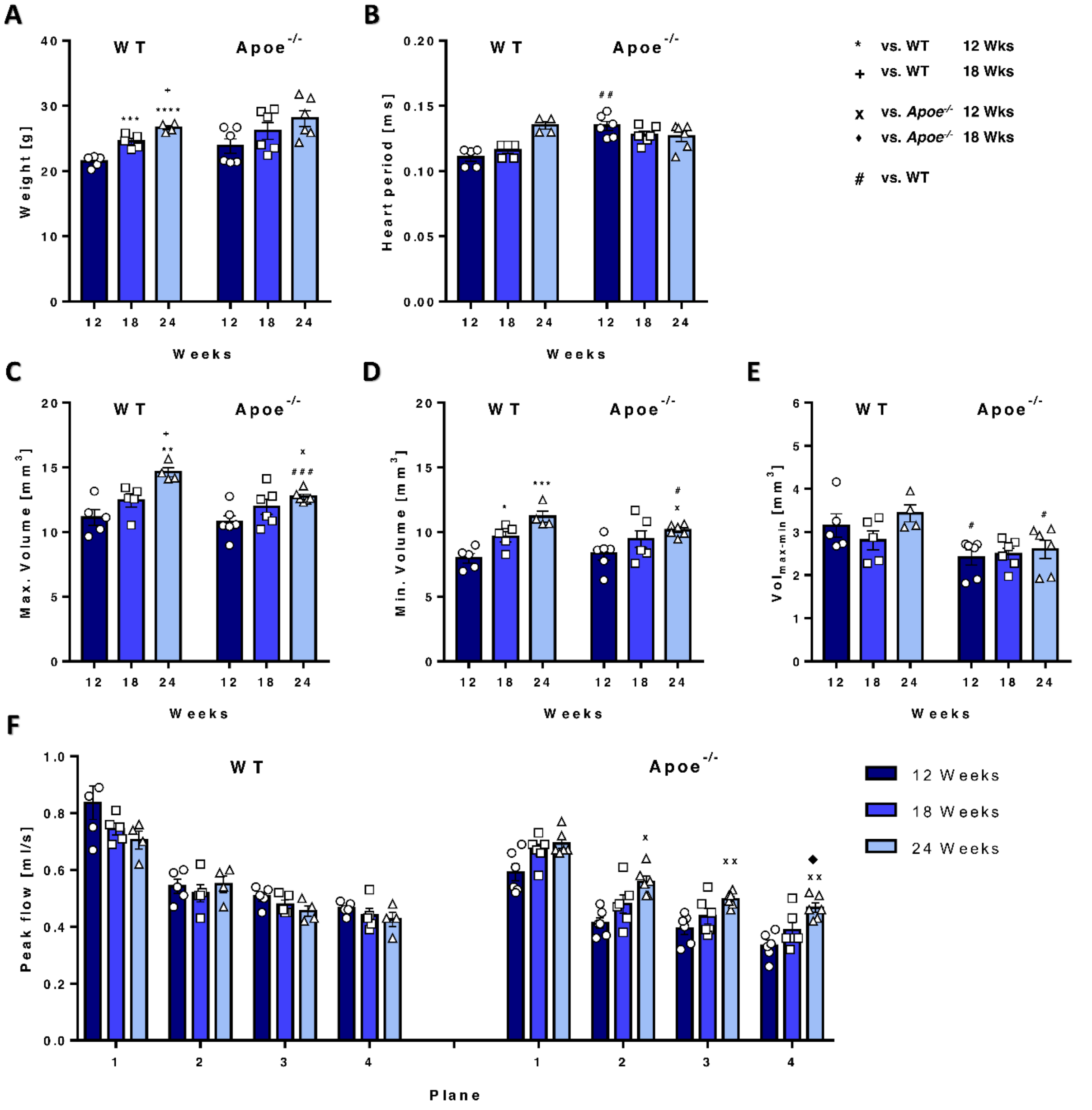


**Supplementary Figure S1: Weight, heart periods, aortic volumes and peak flow of wild type and *Apoe-/-* mice for all measurement timepoints.** **A** WT mice show a significant increase in weight for each time point. No significant increase is observable in the *Apoe-/-* group as well as in comparison to the wildtype group. **B** Heart periods are only different for the first measurement time point at 12 weeks. **C** Maximum aortic volumes. WT mice show a stronger increase in maximum aortic volume over time and significantly larger maxi-mum volumes at 24 weeks compared to *Apoe-/-* mice. **D** Minimum aortic volume. Wildtype mice have a higher increase in mini-mum aortic volume over time and significantly larger minimum volumes at 24 weeks compared to *Apoe-/-* mice. **E** Difference between maximum and minimum aortic volumes. Significant differences can be found at the age of 12 and 24 weeks. **F** Peak flow values in wildtype mice stay constant over time, whereas *Apoe-/-* mice show a significant increase in peak flow over time for analysis planes 2-4.

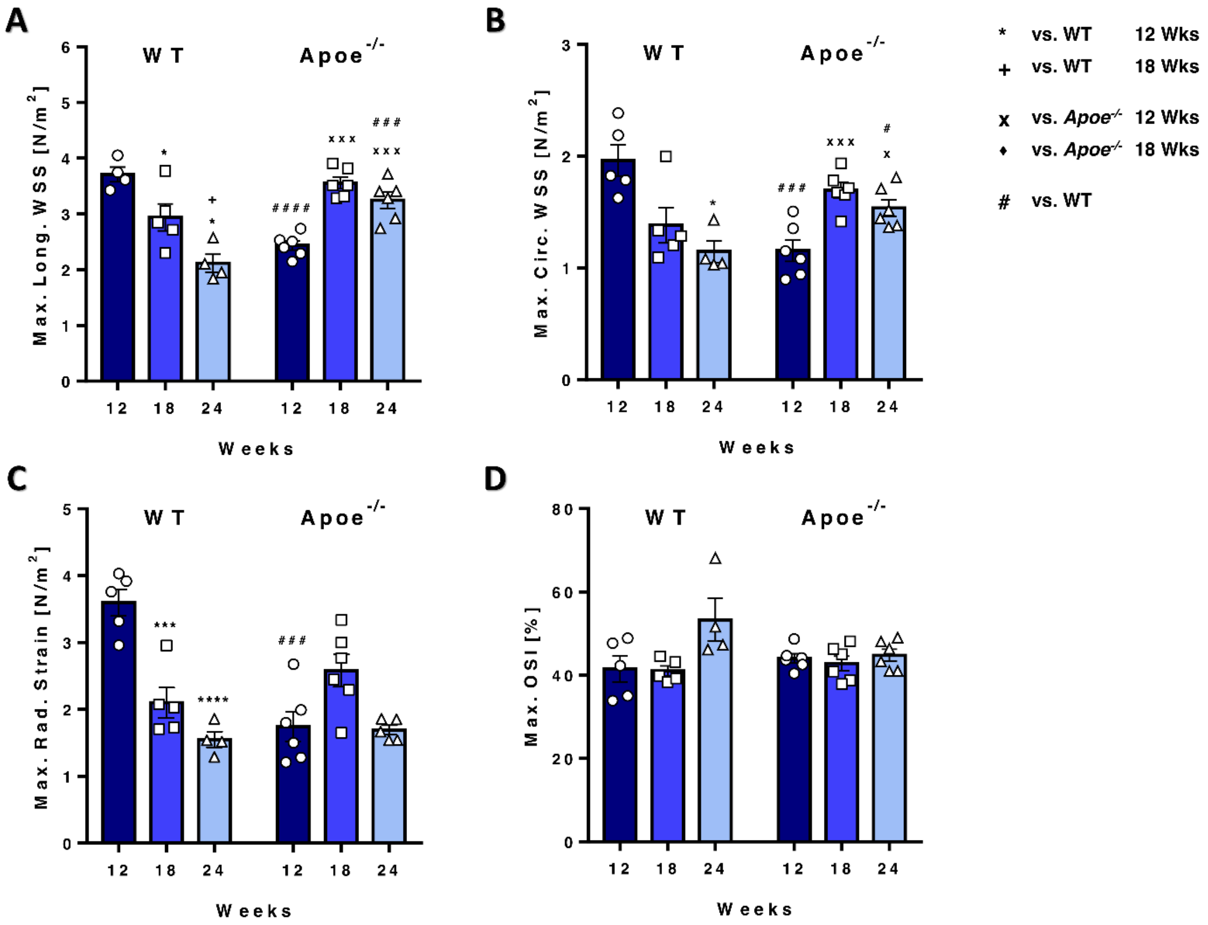


**Supplementary Figure S2: Maximum WSS and OSI values for wildtype and *Apoe*$^{-/-}$ mice over time.** **A** Maximum longWSS values for all measurement time points. WT mice show a significant decrease over time, whereas *Apoe*$^{-/-}$ mice show an increase. Maximum values are furthermore strongly decreased in *Apoe*$^{-/-}$ mice at 12 weeks and increased at 24 weeks compared to WT mice. **B** Maximum circWSS values over time. WT mice show a significant decrease over time, whereas *Apoe*$^{-/-}$ mice show an increase in maximum values over time. Maximum values are furthermore decreased in *Apoe*$^{-/-}$ mice at 12 weeks and increased at 24 weeks compared to WT mice. **C** Maximum radStrain values for all measurement time points. In WT mice, a significant decrease over time is observable, whereas *Apoe*$^{-/-}$ mice show no significant changes. RadStrain values are furthermore significantly decreased in *Apoe*$^{-/-}$ mice at 12 weeks compared to WT mice. **D** Maximum OSI values for all measurement time points. No significant differences are observable.

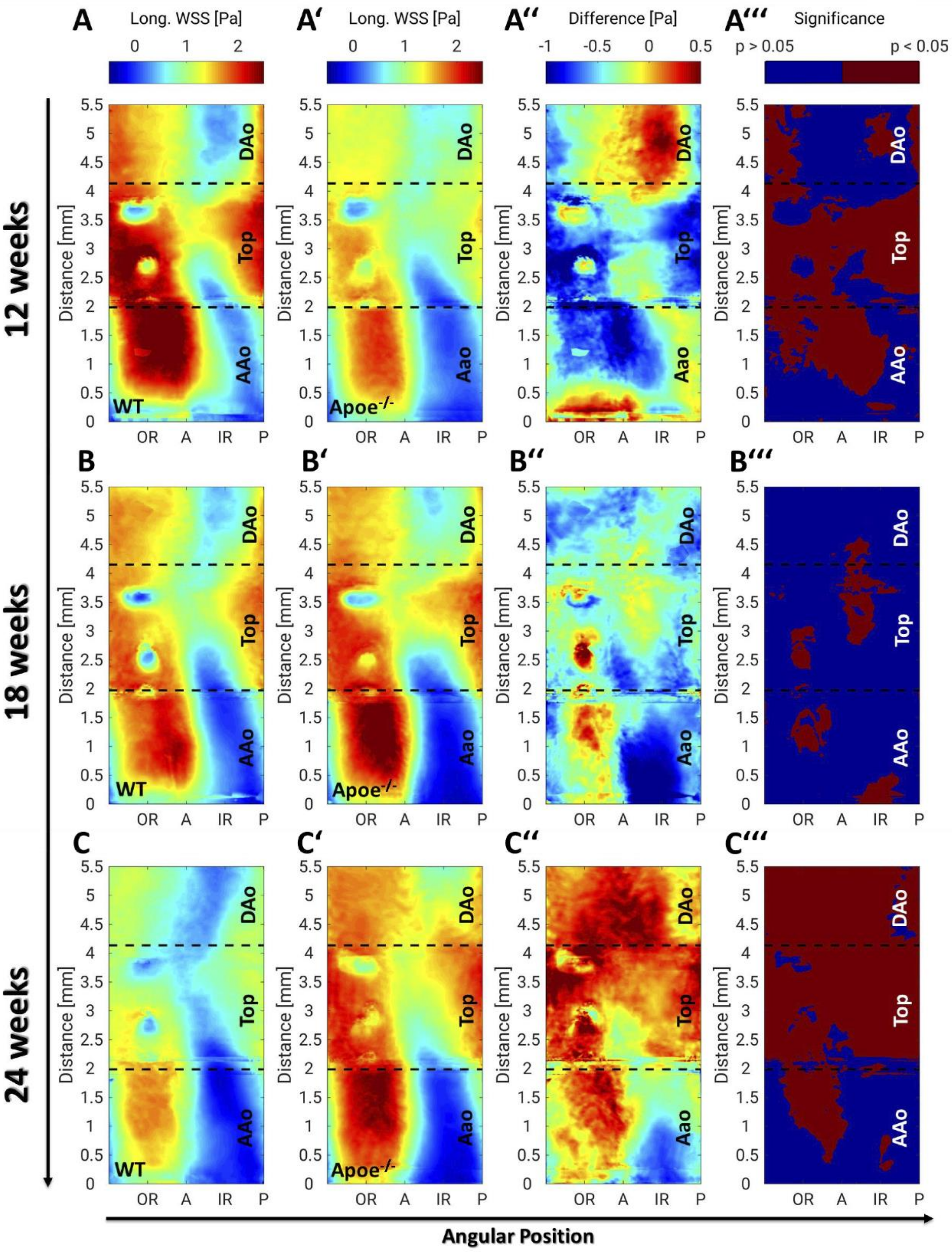


**Supplementary Figure S3: Intergroup comparison of LongWSS for all measurement time points. A** LongWSS map for 12-week-old WT mice and **A'** for *Apoe*$^{-/-}$ mice (group average). **A''** Difference map (*Apoe*$^{-/-}$- WT). **A'''** Significance map. WSS values are significantly increased in the top region, the upper part of the AAo and the posterior side of the DAo in WT mice. A significant difference was also observed near the IR of the DAo. **B** LongWSS map for 18-week-old WT mice and **B'** *Apoe*$^{-/-}$ mice. **B''** Difference map (*Apoe*$^{-/-}$- WT). **B'''** Significance map. Both genotypes show a similar distribution across the arch. **C** LongWSS map for 24-week-old wildtype mice and **C'** for *Apoe*$^{-/-}$ mice. **C''** Difference map (*Apoe*$^{-/-}$- WT). **C'''** Significance map. *Apoe*$^{-/-}$ mice show an overall increase of LongWSS values in the complete top region and DAo.

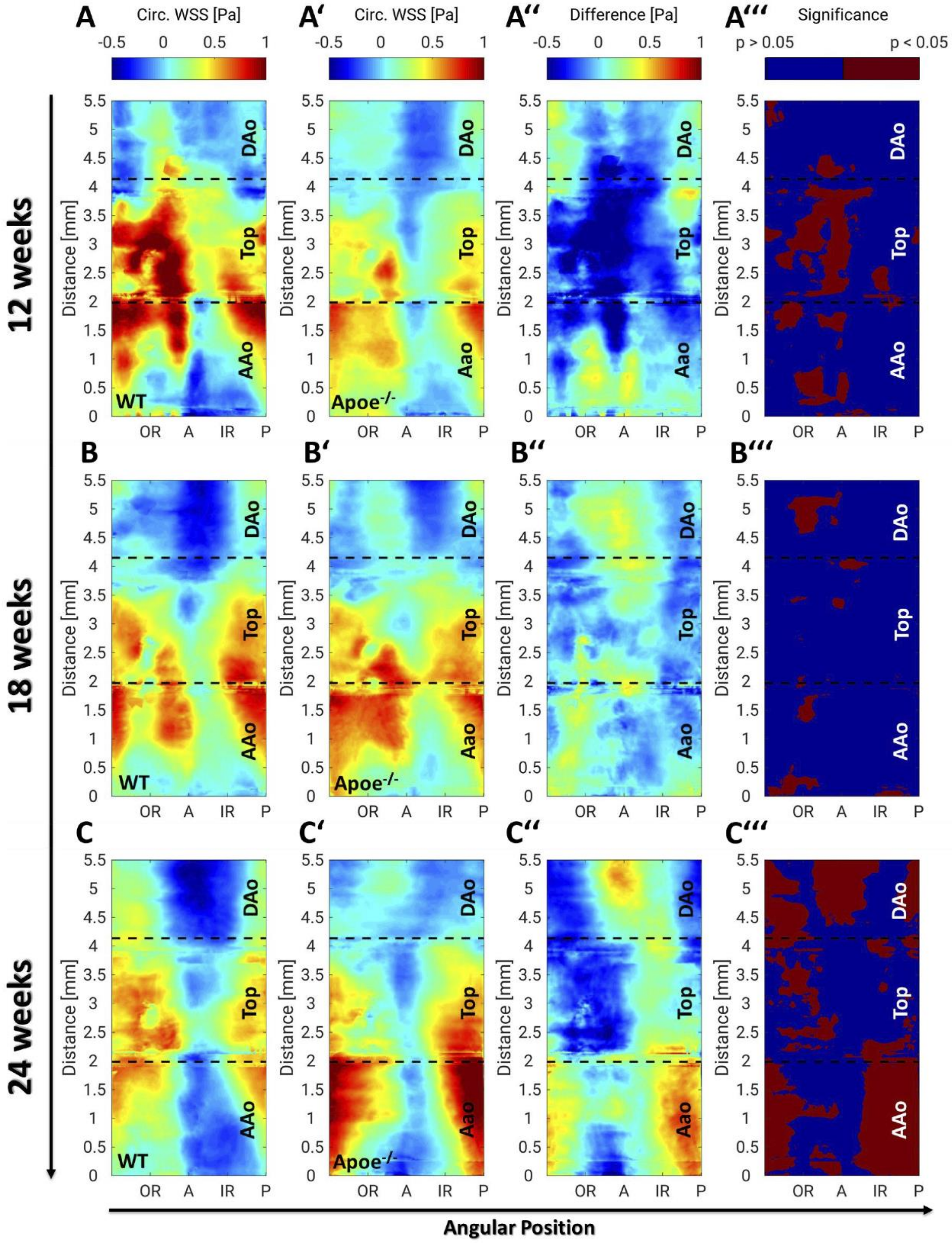


**Supplementary Figure S4: Intergroup comparison of CircWSS for all measurement time points. A** CircWSS map for 12-week-old wildtype mice and **A'** for *Apoe*$^{-/-}$ mice (group average). **A''** Difference map (*Apoe*$^{-/-}$- WT). **A'''** Significance map. CircWSS values of WT mice are significantly increased in the AAo and Top region (from OR to A). **B** CircWSS map for 18-week-old WT mice and **B'** for *Apoe*$^{-/-}$ mice. **B''** Difference map. **B'''** Significance map. Both genotypes show a similar distribution of WSS values across the arch. **C** CircWSS map for 24-week-old WT mice and **C'** for *Apoe*$^{-/-}$ mice. **C''** Difference map. **C'''** Significance map. CircWSS is significantly decreased in WT mice and increased in *Apoe*$^{-/-}$ mice.

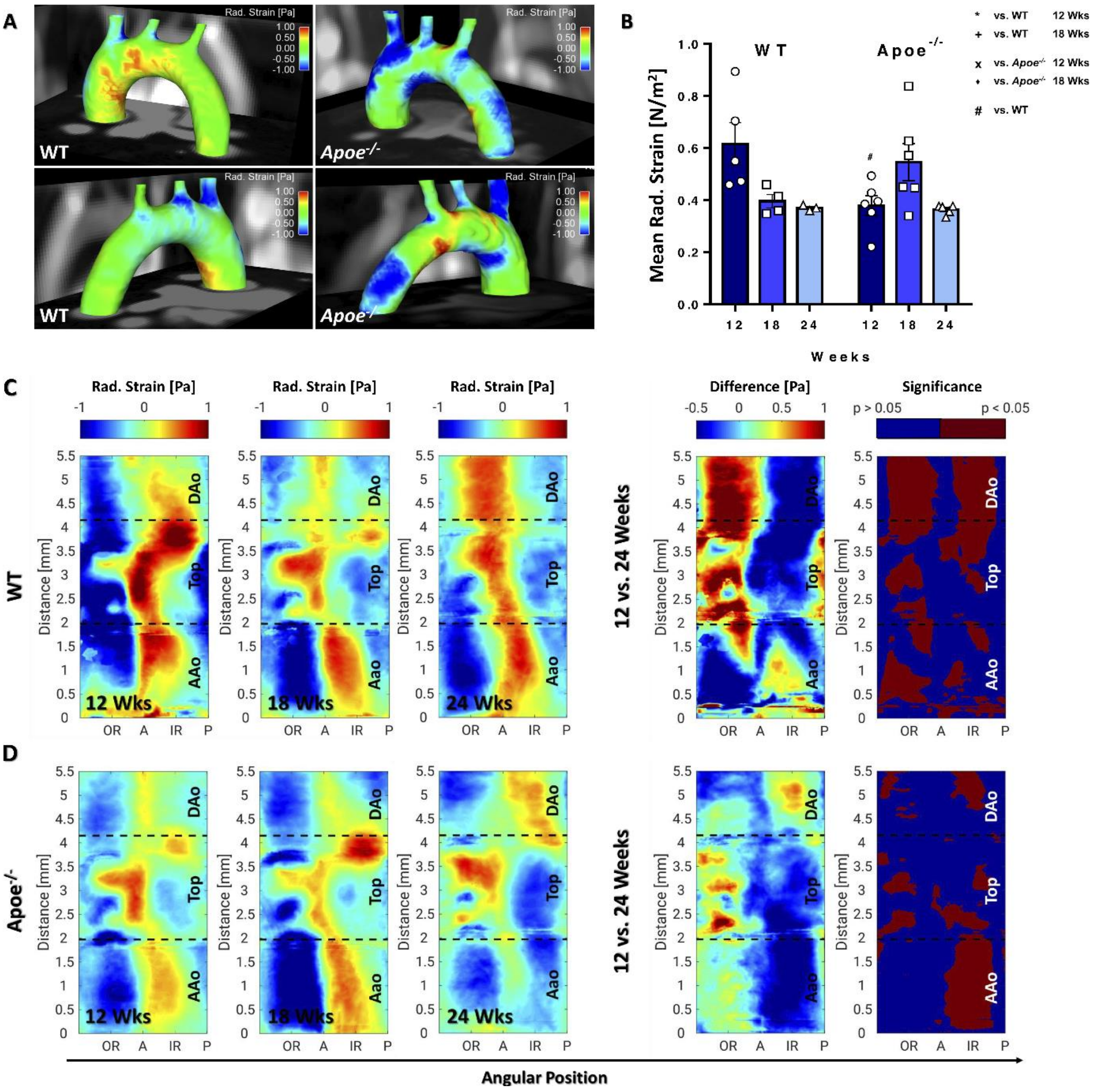


**Supplementary Figure S5: Radial strain shows modest changes in *Apoe*$^{-/-}$ and WT mice over time. A** Three-dimensional radStrain map of a 24-week-old WT mouse (left) and *Apoe*$^{-/-}$ mouse (right) from anterior and posterior view. The wild type and *Apoe*$^{-/-}$ mouse feature significantly different radStrain patterns, especially around the IR and OR of the ascending aorta. **B** Mean radStrain values. Compared to WT mice, a significant decrease in radStrain is observable in *Apoe*$^{-/-}$ mice at 12 weeks. **C** RadStrain maps of WT mice (group average) for all measurement time points and statistical intragroup comparison of radStrain values (12 vs. 24 weeks), showing the difference maps of radStrain values and significance maps. **D** RadStrain Maps of *Apoe*$^{-/-}$ mice.

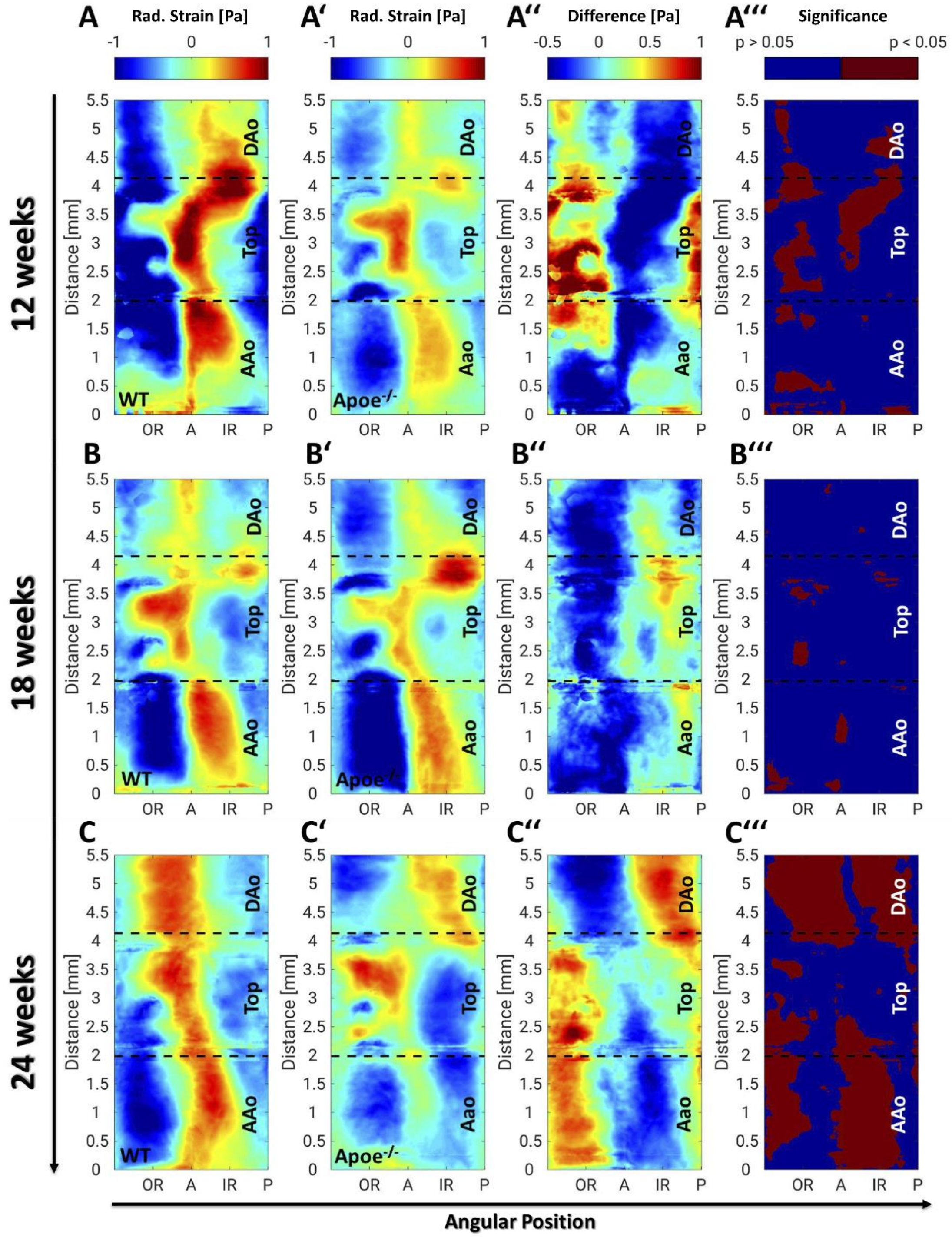


**Supplementary Figure S6: Intergroup comparison of RadStrain for all measurement time points. A** RadStrain map for 12-week-old WT mice and **A'** for *Apoe*$^{-/-}$ mice (group average). **A''** Difference map (*Apoe*$^{-/-}$- WT). **A'''** Significance map. In *Apoe*$^{-/-}$ mice, a significant decrease is visible in the inner and outer radius of the top region. **B** RadStrain map for 18-week-old WT mice and **B'** for *Apoe*$^{-/-}$ mice. **B''** Difference map. **B'''** Significance map. Both genotypes show a similar distribution of radStrain values across the arch. **C** RadStrain map for 24-week-old WT mice and **C'** *Apoe*$^{-/-}$ mice **C''** Difference map. **C'''** Significance map. Large areas of significant differences are visible throughout the complete aortic arch.

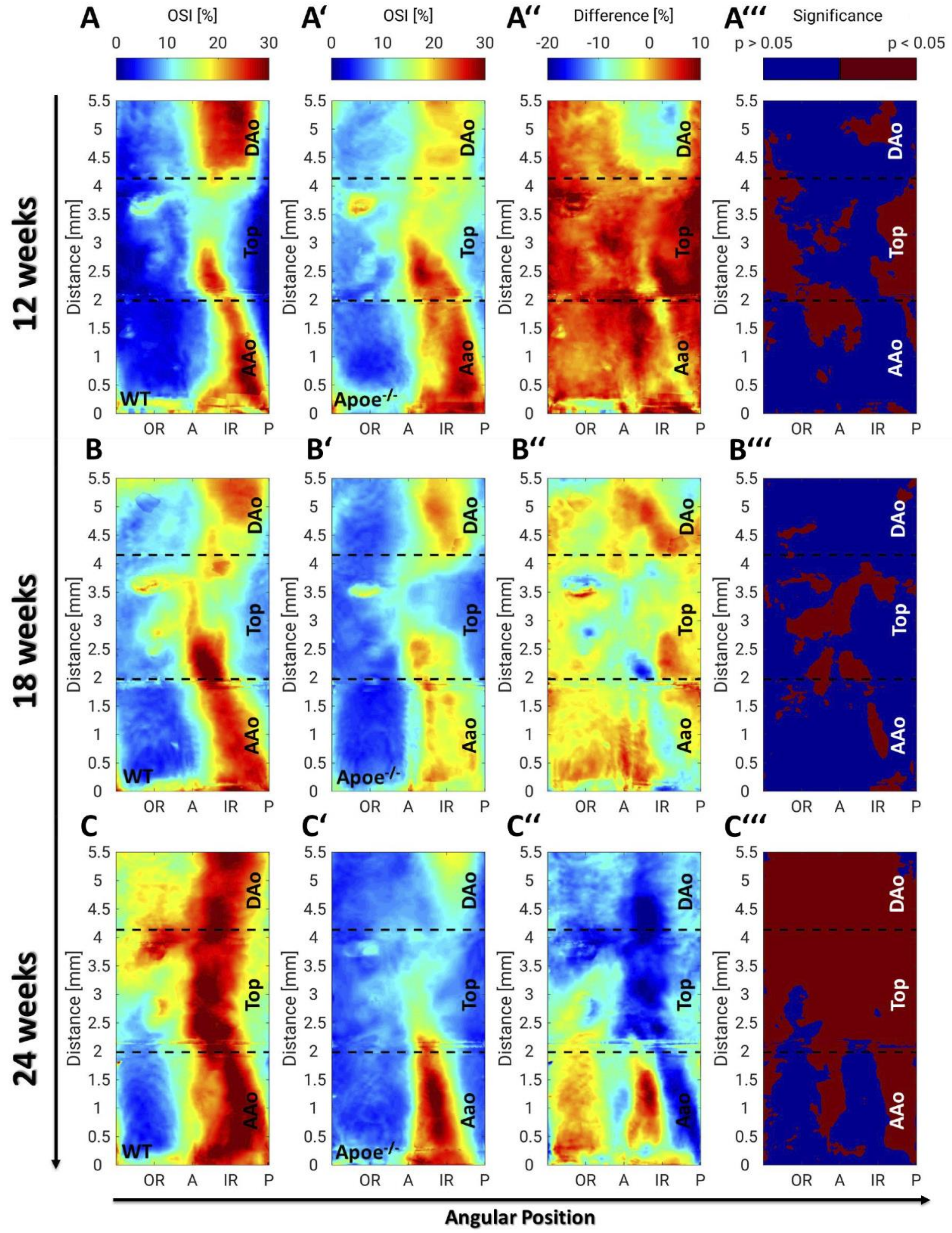


**Supplementary Figure S7: Intergroup comparison of OSI for all measurement time points. A** OSI map for 12-week-old WT mice and **A'** *Apoe*$^{-/-}$ mice (group average). **A''** Difference map (*Apoe*$^{-/-}$- WT). **A'''** Significance map. OSI values are mostly elevated in atherosclerotic mice. **B** OSI map for 18-week-old WT mice and **B'** *Apoe*$^{-/-}$ mice. B'' Difference map. **B'''** Significance map. OSI values are elevated around the inner radius of WT mice and significantly lower near the inner radius of atherosclerotic mice. **C** OSI map for 24-week-old WT mice and **C'** *Apoe*$^{-/-}$ mice. **C''** Difference map. **C'''** Significance map. A significant decrease in OSI values can be observed in the top region and descending aorta in *Apoe*$^{-/-}$ mice.

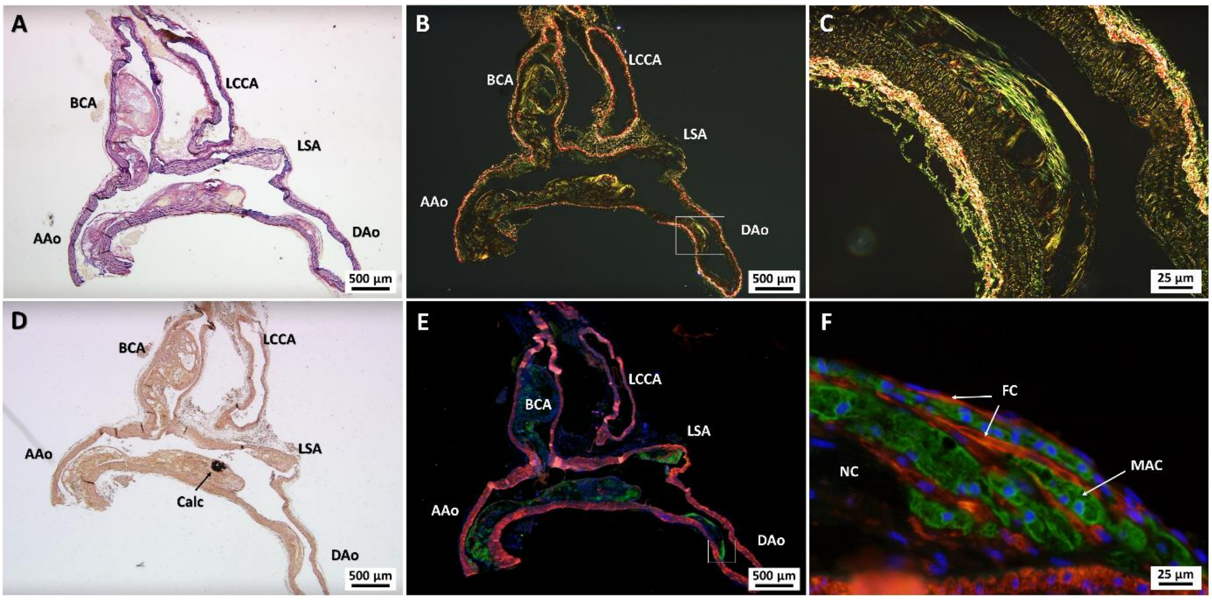


**Supplementary Figure S8: Histological stainings of longitudinal sections of an exemplary *Apoe*$^{-/-}$ mouse. A** Aldehyde-Fuchsin overview staining. **B** Picro-siriusred staining under polarized light for collagen analysis. **C** Enlarged view of **B** (see white Box). D Silvering after Kossa staining for calcification analysis (see black arrow). **E** Immunofluorescence staining of MAC-2 for macrophages (green) and aSMA for smooth muscle cells (red). Nuclei are counterstained with DAPI (blue). **F** Enlarged view of E (see white box) with visible fibrous cap (FC), formed by SMCs, necrotic core (NC) and foam cells (MAC).
