# Supplementary material for: 2D Projection Maps of WSS and OSI Reveal Distinct Spatiotemporal Changes in Hemodynamics in the Murine Aorta during Ageing and Atherosclerosis
Source: Biomedicines. 2021 Dec 7;9(12):1856. doi: 10.3390/biomedicines9121856 (PMC8698968; doi:10.3390/biomedicines9121856)
Supplement: Supplementary file 1 [file biomedicines-09-01856-s001.zip › Supplementary Tables.pdf]

Supplementary Tables

Supplementary Table S1: Spatial median, lower quartile and upper quartile values of the intragroup standard deviations (STD) in wild type mice, determined from the pixel values of the 2D projection maps. The respective highest median values are marked in bold.

Long. WSS STD	Median value [Pa]	Lower Quartile [Pa]	Upper Quartile [Pa]
Weeks			
12	**0.34**	0.25	0.45
18	0.29	0.22	0.39
24	0.19	0.13	0.26
**Circ. WSS STD**	**Median value [Pa]**	**Lower Quartile [Pa]**	**Upper Quartile [Pa]**
Weeks			
12	**0.30**	0.19	0.40
18	0.29	0.22	0.39
24	0.12	0,08	0.17
**Rad Strain STD**	**Median value [Pa]**	**Lower Quartile [Pa]**	**Upper Quartile [Pa]**
Weeks			
12	**0.47**	0.33	0.67
18	0.29	0.22	0.39
24	0.23	0.14	0.35
**OSI STD**	**Median value [%]**	**Lower Quartile [%]**	**Upper Quartile [%]**
Weeks			
12	4.6	3.2	6.6
18	**5.8**	3.7	8.4
24	4.4	2.4	6.8

Supplementary Table S2: Spatial median, lower quartile and upper quartile values of the intra group standard deviations (STD) in *Apoe*$^{-/-}$ mice, determined from the pixel values of the 2D projection maps. The respective highest median values are marked in bold.

**Long. WSS STD**	**Median value [Pa]**	**Lower Quartile [Pa]**	**Upper Quartile [Pa]**
Weeks			
12	0.27	0.20	0.36
18	**0.39**	0.28	0.54
24	0.33	0.23	0.47
**Circ. WSS STD**	**Median value [Pa]**	**Lower Quartile [Pa]**	**Upper Quartile [Pa]**
Weeks			
12	0.20	0.16	0.26
18	**0.25**	0.16	0.35
24	0.21	0.12	0.30
**Rad Strain STD**	**Median value [Pa]**	**Lower Quartile [Pa]**	**Upper Quartile [Pa]**
Weeks			
12	0.33	0.26	0.42
18	**0.50**	0.34	0.71
24	0.29	0.20	0.38
**OSI STD**	**Median value [%]**	**Lower Quartile [%]**	**Upper Quartile [%]**
Weeks			
12	**6.3**	4.8	7.8
18	5.5	3.4	7.9
24	3.4	2.1	6.2
