# Supplementary figures and images for: 2D Projection Maps of WSS and OSI Reveal Distinct Spatiotemporal Changes in Hemodynamics in the Murine Aorta during Ageing and Atherosclerosis
Source: Biomedicines. 2021 Dec 7;9(12):1856. doi: 10.3390/biomedicines9121856 (PMC8698968; doi:10.3390/biomedicines9121856)

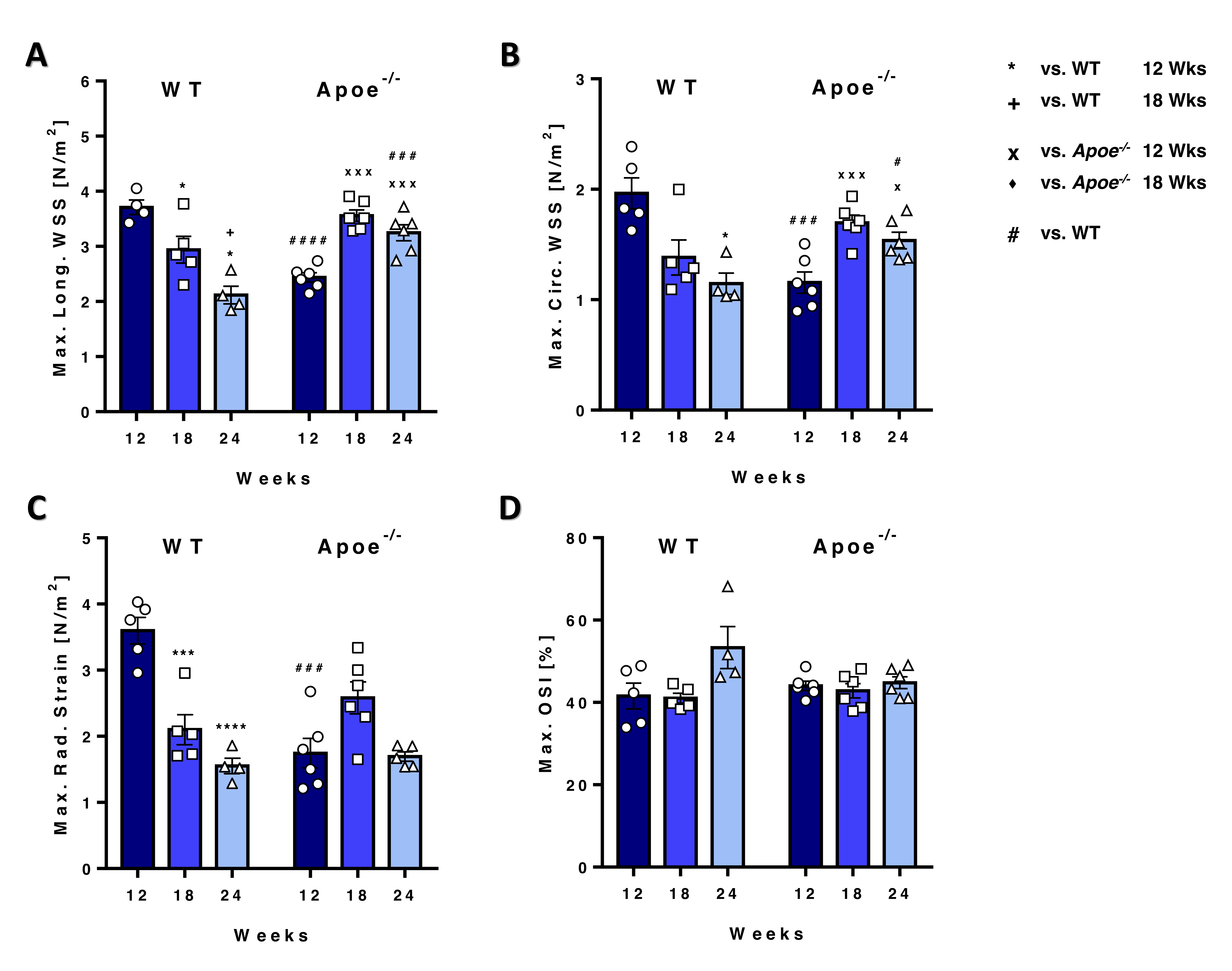

Supplement: Supplementary file 1 [file biomedicines-09-01856-s001.zip › Supplementary_Figure_2.tiff]

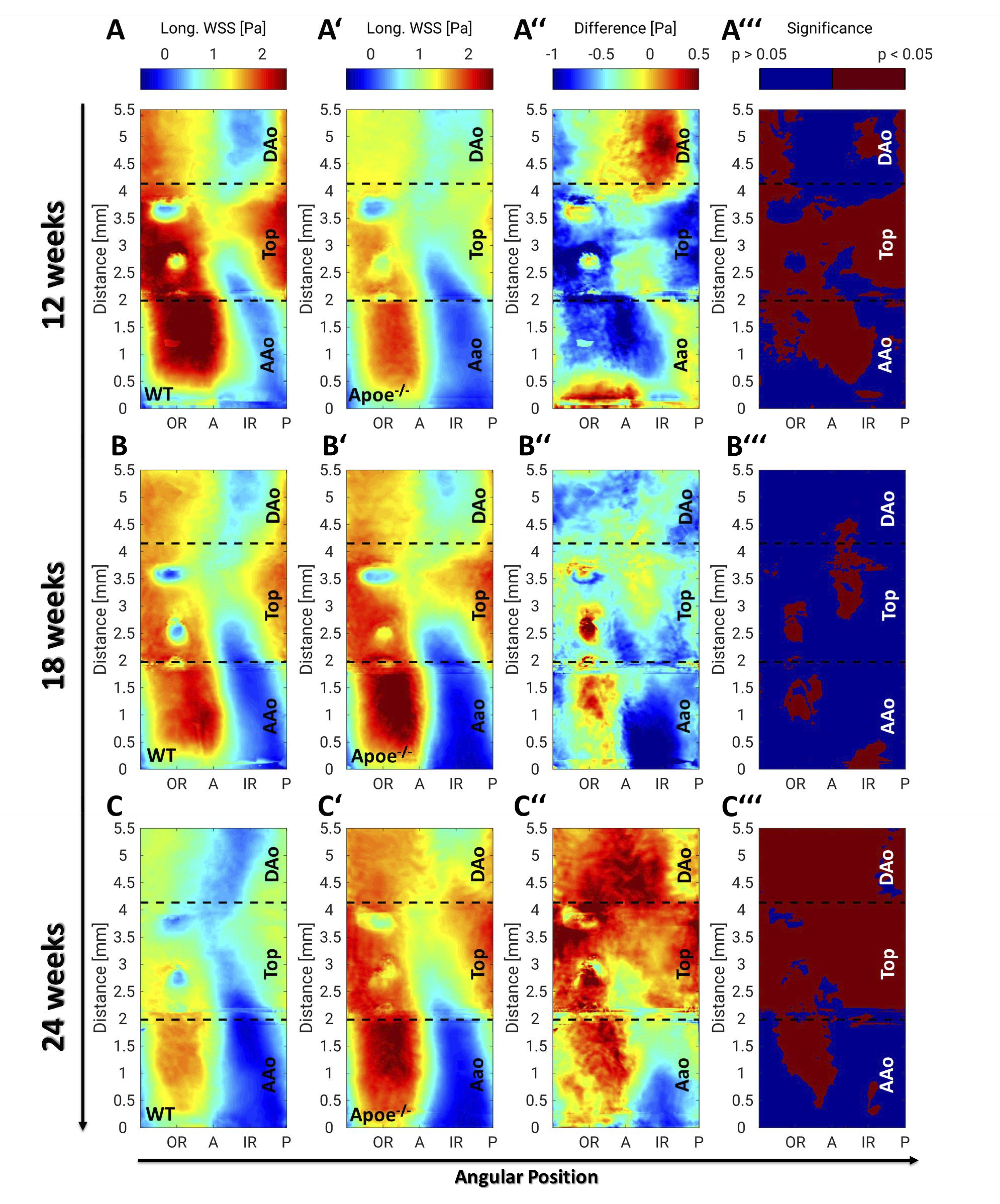

Supplement: Supplementary file 1 [file biomedicines-09-01856-s001.zip › Supplementary_Figure_3.tiff]

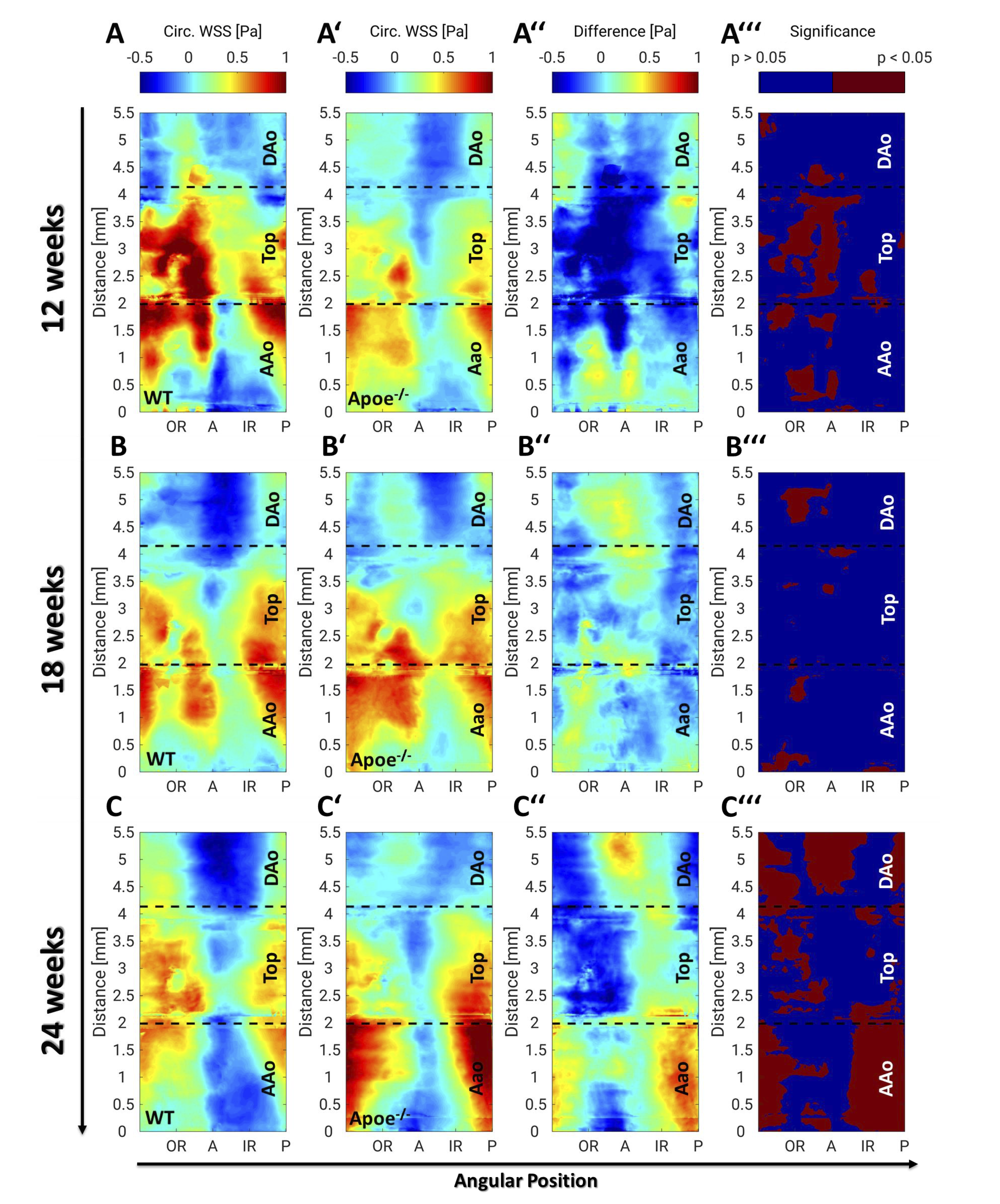

Supplement: Supplementary file 1 [file biomedicines-09-01856-s001.zip › Supplementary_Figure_4.tiff]

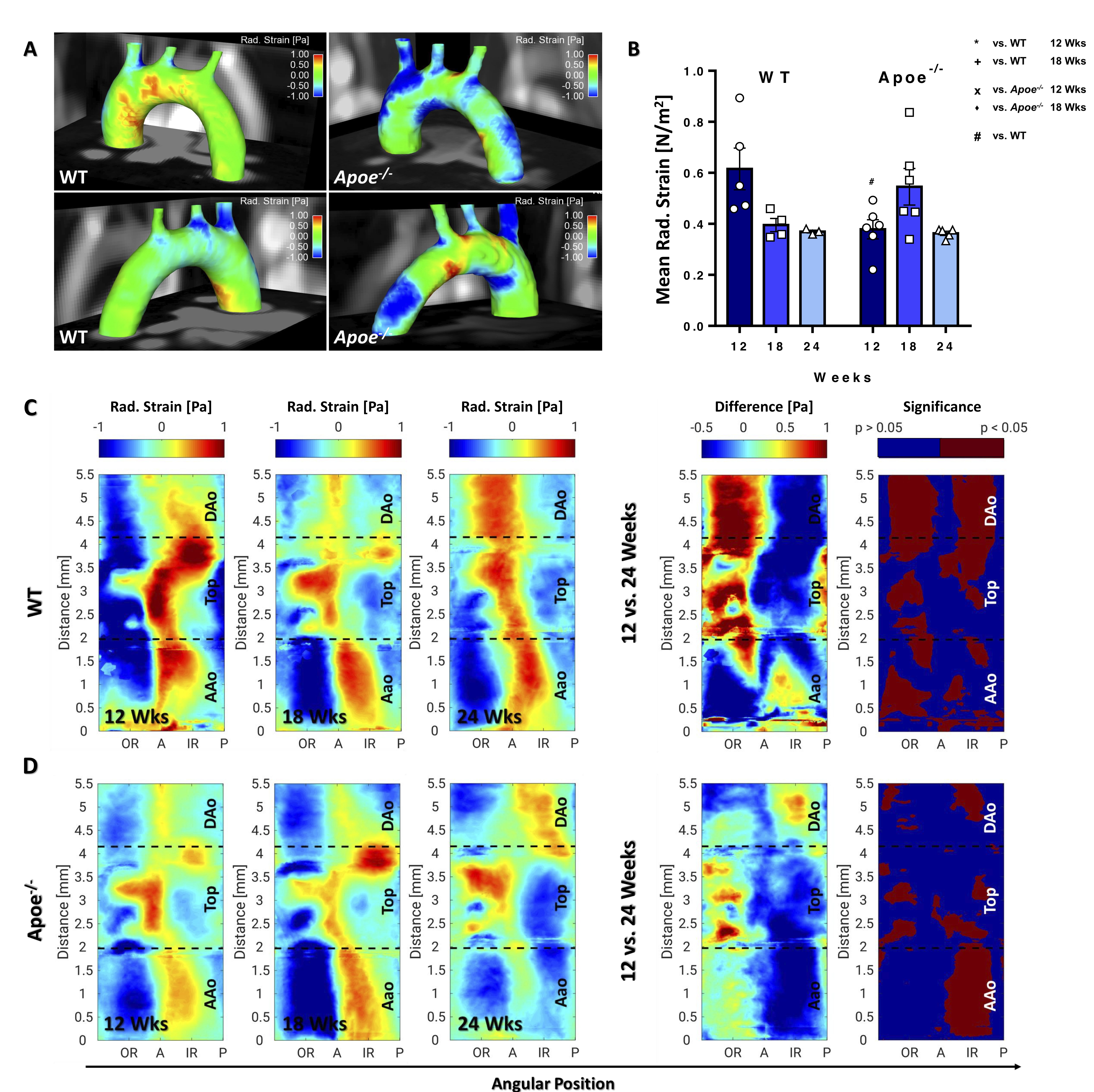

Supplement: Supplementary file 1 [file biomedicines-09-01856-s001.zip › Supplementary_Figure_5.tiff]

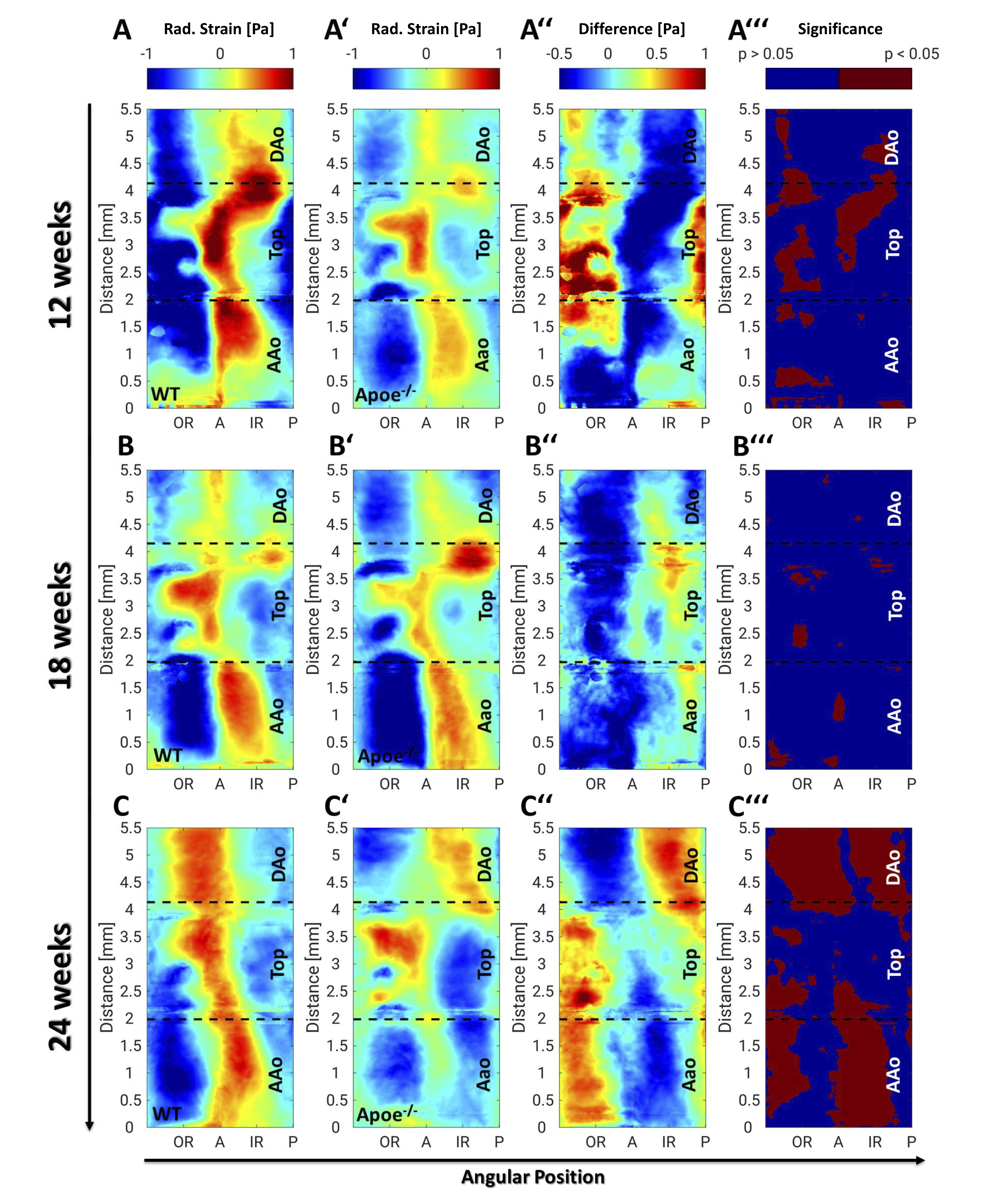

Supplement: Supplementary file 1 [file biomedicines-09-01856-s001.zip › Supplementary_Figure_6.tiff]

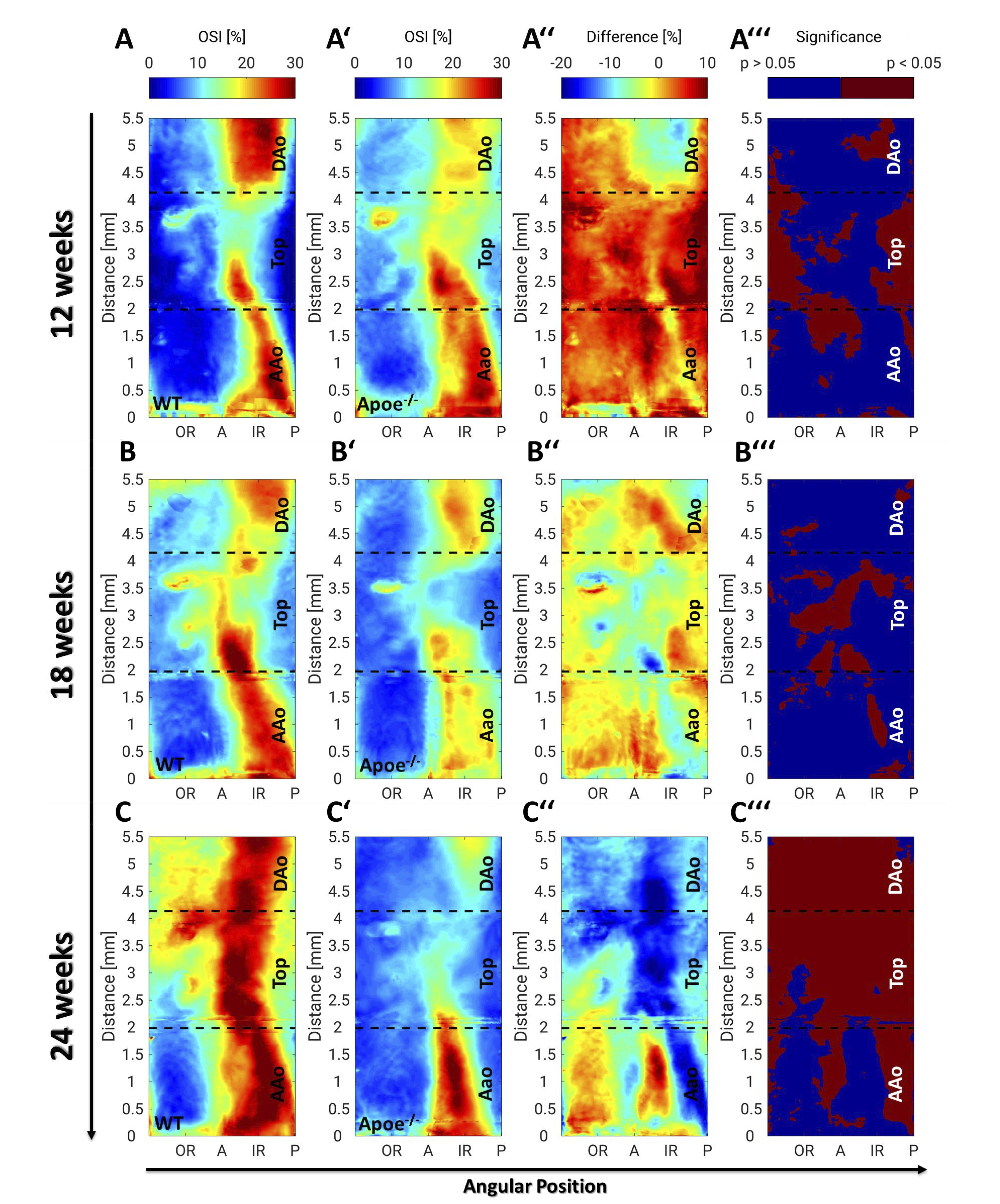

Supplement: Supplementary file 1 [file biomedicines-09-01856-s001.zip › Supplementary_Figure_7.tiff]

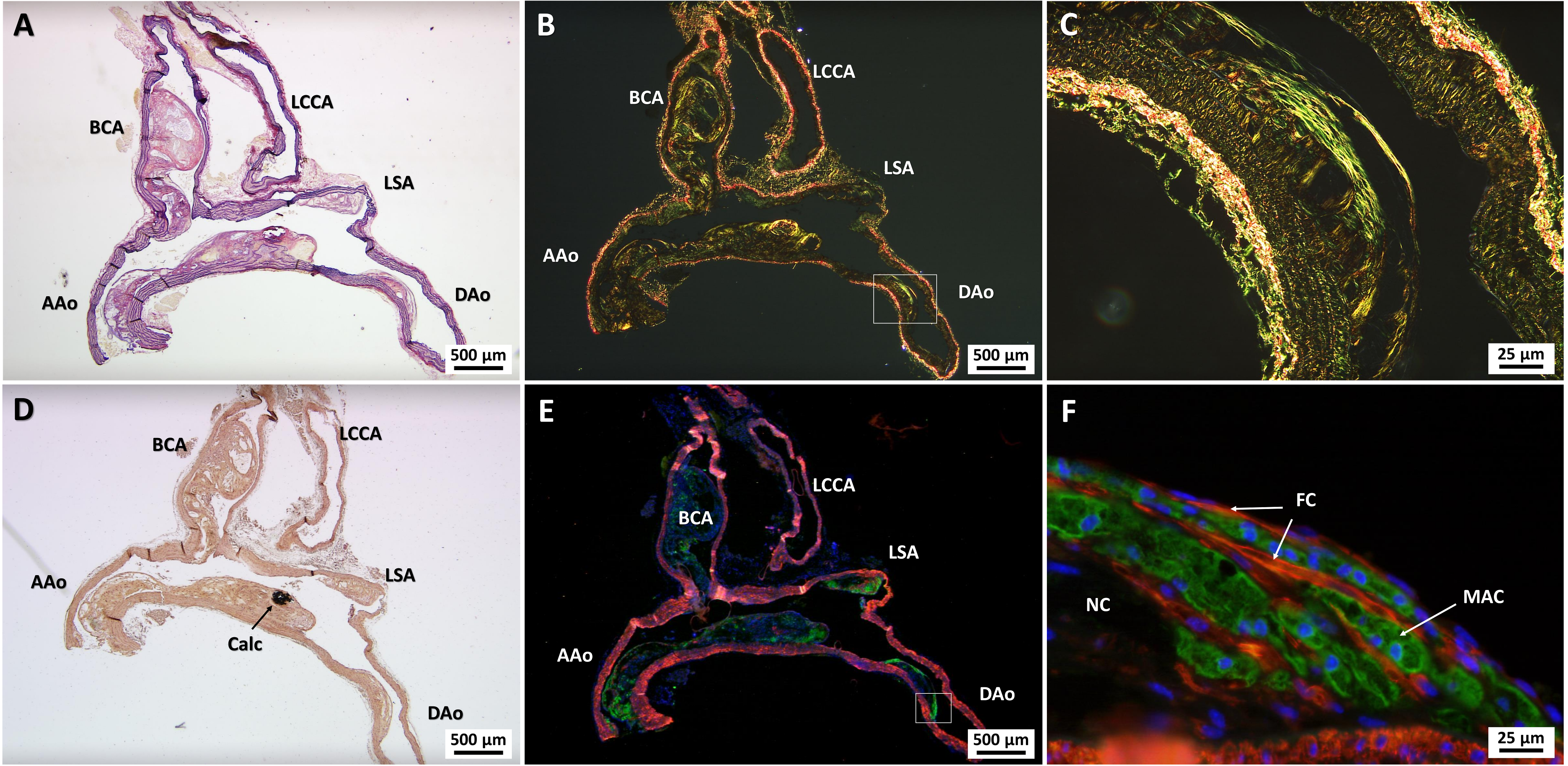

Supplement: Supplementary file 1 [file biomedicines-09-01856-s001.zip › Supplementary_Figure_8.tif]
